# Contributions of de novo variants to systemic lupus erythematosus

**DOI:** 10.1038/s41431-020-0698-5

**Published:** 2020-07-28

**Authors:** Jonas Carlsson Almlöf, Sara Nystedt, Aikaterini Mechtidou, Dag Leonard, Maija-Leena Eloranta, Giorgia Grosso, Christopher Sjöwall, Anders A. Bengtsson, Andreas Jönsen, Iva Gunnarsson, Elisabet Svenungsson, Lars Rönnblom, Johanna K. Sandling, Ann-Christine Syvänen

**Affiliations:** 1 Department of Medical Sciences, Molecular Medicine and Science for Life Laboratory, Uppsala University, 751 23 Uppsala, Sweden; 2 Department of Medical Sciences, Rheumatology and Science for Life Laboratory, Uppsala University, 751 85 Uppsala, Sweden; 3 Department of Medicine, Karolinska Institutet, Rheumatology, Karolinska University Hospital, 171 77 Stockholm, Sweden; 4 Department of Clinical and Experimental Medicine, Rheumatology/Division of Neuro and Inflammation Sciences, Linköping University, 581 83 Linköping, Sweden; 5 Department of Clinical Sciences, Rheumatology, Lund University, Skåne University Hospital, 222 42 Lund, Sweden

**Keywords:** Systemic lupus erythematosus, Genetics research

## Abstract

By performing whole-genome sequencing in a Swedish cohort of 71 parent-offspring trios, in which the child in each family is affected by systemic lupus erythematosus (SLE, OMIM 152700), we investigated the contribution of de novo variants to risk of SLE. We found de novo single nucleotide variants (SNVs) to be significantly enriched in gene promoters in SLE patients compared with healthy controls at a level corresponding to 26 de novo promoter SNVs more in each patient than expected. We identified 12 de novo SNVs in promoter regions of genes that have been previously implicated in SLE, or that have functions that could be of relevance to SLE. Furthermore, we detected three missense de novo SNVs, five de novo insertion-deletions, and three de novo structural variants with potential to affect the expression of genes that are relevant for SLE. Based on enrichment analysis, disease-affecting de novo SNVs are expected to occur in one-third of SLE patients. This study shows that de novo variants in promoters commonly contribute to the genetic risk of SLE. The fact that de novo SNVs in SLE were enriched to promoter regions highlights the importance of using whole-genome sequencing for identification of de novo variants.

## Introduction

Systemic lupus erythematosus (SLE, OMIM 152700) is a chronic autoimmune disease that affects multiple organs of the human body. SLE is ninefold enriched among women and has an estimated prevalence in the Swedish population of 68 in 100,000 [1] and prevalence estimates vary between 20 and 100 per 100,000 in other European populations [2]. In the past decade, genome-wide association studies have identified more than 100 risk loci that are associated with SLE [3, 4] and account for ~30% of the genetic susceptibility of SLE [5]. Mutations causing monogenic SLE that contribute to the heritability of the disease have been detected in 1–3% of SLE patients [6]. In an earlier study we have shown that ultra-rare missense variants in the same genes as those causing monogenic forms of SLE make an additional contribution to the heritability of SLE [7]. De novo variants (DNVs) could be an alternative mechanism that increases the burden of genetic risk in SLE. De novo mutations occur during the formation of the gametes and are thereby not inherited from the parents, although they are present in every cell of the child. On average 70 DNVs are expected to be introduced per human generation, and out of them one to two DNVs affect the protein-coding sequence [8]. Using whole-genome sequencing (WGS) of parent-offspring trios both inherited mutations and DNVs can be detected.

Most studies on DNVs in complex diseases have focused on developmental disorders like autism spectrum disorder and schizophrenia [9], but also congenital heart defects have been found to be frequently caused by DNMs [10]. A majority of the sporadic cases that receive a molecular diagnosis by clinical sequencing can be fully explained by de novo variants [11]. It has also been shown that the genomes of individuals with severe undiagnosed developmental disorders carry an excess of DNVs in developmentally important genes. Based on exome sequencing it has been estimated that more than 40% of children with developmental diseases or syndromes carry deleterious DNVs in their protein-coding sequences [12]. In addition, an enrichment of DNVs with incomplete penetrance has been observed in individuals with milder forms of autism [13]. DNVs have also been implicated in certain sporadic cancers of young individuals [14].

As there is no selection pressure on non-lethal DNVs, the probability that the DNVs cause increased disease risk is higher than for inherited variants in a population. The contribution of DNVs to genetic risk in complex diseases like SLE has not been investigated in depth, but as SLE is typically caused by a combination of multiple genetic risk factors with small effect sizes, the disease contribution by SLE can be expected to be lower than for early-onset developmental disorders.

The investigations of DNVs in autoimmune diseases have so far focused on individuals with early-onset and severe cases of the disease [15], where the effects of de novo variants are presumed to be large and thus easier to identify than in patients with the more common complex disease etiology. To identify DNVs in SLE, an early study used exome sequencing of 30 early-onset SLE patients with extreme-phenotype, but this study only confirmed one de novo variant, in addition to 13 potential missense DNV in genes that are not expected to increase the risk of SLE [16]. An unbiased and comprehensive investigation of the contribution of DNVs to SLE and other autoimmune disease still remains to be performed.

## Results

In this study the goal was to identify de novo single nucleotide variants (SNVs), de novo insertion-deletions (INDELs) and de novo structural variants (SVs) in trio families with a child affected by SLE and to determine the potential contribution of de novo variants to the genetic risk of SLE. We investigated the contribution of de novo variants to SLE using WGS of 71 parent-offspring trios.

### Frequency and origin of de novo single nucleotide variants in SLE

By using WGS to generate 30x sequencing coverage of the genomes of 213 individuals from 71 trio families, followed by calling of SNVs, stringent filtering, and experimental validation of a subset of de novo SNVs by the polymerase chain reaction, followed by Sanger sequencing, we detected a total of 4157 de novo SNVs that were present in the genomes of the child, but absent from the parents’ genomes.

The total number of de novo SNVs detected in 71 SLE patients corresponds to on average 59 de novo SNVs per SLE patient (range 40–91). This number compares well with the rate of 73 de novo SNVs detected per healthy individual in the Danish population using WGS combined with de novo assembly of the genomes of 25 healthy trio families [17], and with the data from a large study of 1548 trio families from the Icelandic population, which identified 65 de novo SNVs per trio [8]. Thus we do not find evidence in our data that the total number of de novo SNVs per individual would be overrepresented in SLE compared with controls.

The parent of origin of de novo SNVs in the offspring was determined and, as expected, due to the continued cell divisions in sperm cells after puberty, the number of de novo SNVs originating from the father was almost four times higher (*n* = 269) than the number of de novo SNVs with a maternal origin (*n* = 73). The effect of the age at conception of the father in our study was calculated to cause an increase of 1.56 de novo variants per year in the child affected with SLE. This value corresponds to a correlation coefficient of 0.49 (*p* value = 2.97E–7) between age of the father and the rate of de novo variants (Supplementary Fig. S1). When conditioning the linear regression on the age of the mother, each year in the age of the father adds 1.34 de novo variants (*p* value 0.00158).

### Enrichment of de novo SNVs to promoter regions in SLE patients

**Fig. 1:**
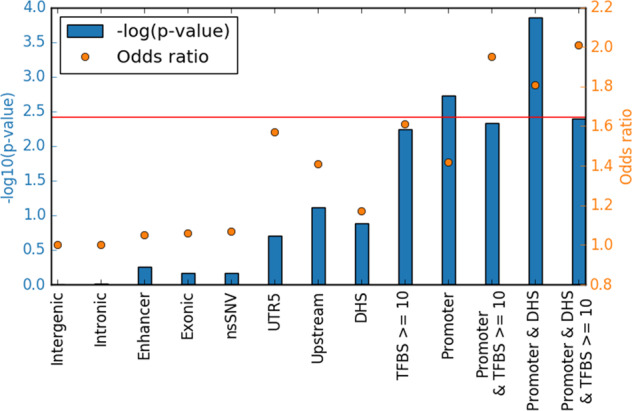
Odds ratios and *p* values. Level of overrepresentation and significance of de novo SNVs in functionally annotated genomic regions. Red line indicates significance at a Bonferroni corrected *p* value of 0.05 (corrected for 13 tests). DHS = DNAse I hypersensitive site indicating open chromatin, TFBS transcription factor binding site.

Next we investigated whether de novo SNVs in SLE were enriched or depleted in specific genomic contexts. Comparison of the genomic distribution of de novo SNVs in the SLE patients in our study with that of healthy individuals from an Icelandic study [8] allowed identification of regions of the genome that are differentially affected by de novo SNVs in SLE. These regions could potentially be involved in the pathogenesis of SLE. For SNVs in functionally inactive regions, an odds ratio of 1.0 is expected, which is in line with an odds ratio of 1.0 that we observe for the de novo SNVs in functionally inactive intronic and intergenic regions (Fig. 1).

Compared with the de novo variants in the data from the healthy individuals we observed a suggestive enrichment of de novo SNVs in 5′-untranslated regions (UTR5) and regions upstream of gene promoters (Table 1, Fig. 1), while de novo variants in SLE patients were not enriched in exons and enhancers. By annotation of the variants based on predicted chromatin states [18] in B lymphocytes, we observed a significant enrichment of de novo SNVs in promoters in the SLE patients compared with healthy individuals (OR = 1.42, *p* value 0.0019, Pearson’s chi-squared test). B cells are of particular interest in SLE as the disease is characterized by B cell dysfunction [19]. The signal of enrichment was further strengthened by overlapping the DNV annotated to promoter regions with DHS regions of open chromatin (1.81, *p* value 0.00014). Small additional gains in odds ratio were also observed by adding a filter on variants that overlap more than 10 transcription factor binding sites (TFBS) (Table 1, Fig. 1, and Supplementary Fig. S2).

**Table 1:** Enrichment of de novo SNVs in SLE patients to functionally annotated genomic regions.

Feature	No of SNVs in SLE trios	No of SNVs in healthy trios [10]	Odds ratio	*P* value	Expected no of potential disease related SNVs based on the odds ratio^a^	Identified potential diseaserelated SNVs reported in Table 2
Gene position						
UTR5	11 (0.26%)	165 (0.17%)	1.57	0.20	4	2
Upstream of gene	32 (0.77%)	536 (0.55%)	1.41	0.077	9	4
Exon	69 (1.66%)	1535 (1.57%)	1.06	0.69	–	–
Intron	1543 (37.1%)	36,426 (37.2%)	1.00	0.96	–	–
Intergenic region	2197 (52.9%)	51,674 (52.8%)	1.00	0.96	–	–
Chromatin state^b^						
Promoter	87 (2.09%)	1442 (1.47%)	1.42	0.0019*	26	12
Enhancer	210 (5.05%)	4724 (4.82%)	1.05	0.55	–	–
Other annotations						
nsSNV	51 (1.23%)	1122 (1.15%)	1.07	0.69	–	–
No of TFBS > = 10	39 (0.94%)	573 (0.59%)	1.61	0.0057	15	8
Promoter + TFBS > = 10	21 (0.51%)	254 (0.26%)	1.95	0.0047	10	8
DHS	109 (2.62%)	2194 (2.24%)	1.17	0.13	16	12
Promoter + DHS	46 (1.11%)	599 (0.61%)	1.81	0.00014*	21	12
Promoter + DHS+ TFBS > = 10	20 (0.48%)	235 (0.24%)	2.01	0.0040	10	8

*Significant after Bonferroni correction (*n* = 13).

*UTR5* 5′-untranslated region, *nsSNV* non-synonymous single nucleotide variant, *TFBS* transcription factor binding site, *DHS* DNase I hypersensitive site indicating open chromatin.

^a^Expected number of disease related SNVs = Number of SNVs–(Number of SNVs/odds ratio).

^b^Prediction of chromatin state based on ChIP-seq histone modification peak data in B cells using ChromHMM).

### De novo SNVs in promoters with potential to affect risk of SLE

The genes located closest to the de novo SNVs identified in promoter regions of the SLE genomes were investigated further regarding their putative roles as risk factors for SLE. Of the 87 de novo promoter SNVs only two were annotated to the same genes (*ZNF292*). Pathway analysis of these genes using Gene Set Enrichment Analysis and the Genotype-Tissue Expression dataset revealed no significant pathways.

Each of 33 of the 87 de novo SNVs annotated to a promoter region were judged to have large potential to affect transcription factor binding due its their central position in relation to a large number of TFBS, its closeness to a sequence peak of active histone marks and overlap with a region of open chromatin in B-lymphocytes [20]. Indeed, among these 33 SNVs, 25 were located close to genes with differential gene expression between SLE patients and controls, according to data in the gene expression omnibus (GEO) database (FDR-corrected *p* value < 0.01, Supplementary Table S1) [21, 22], which compared with a random selection of de novo SNVs with matching properties and annotations in the healthy controls, this represents a significant enrichment (OR 1.66, *p* value 0.0157).

By literature search of the genes close to the 33 de novo SNVs located in central parts of a promoter we found twelve genes with known functions that could be relevant for SLE directly or indirectly through involvement in autoimmunity, apoptosis, or inflammation (Table 2, Supplementary Table S2, Supplementary Fig. S3). None of the genes reported in Table 2 have any similarly located de novo SNVs in the Icelandic controls. We also identified 9 additional de novo SNVs with high potential to affect the expression of a nearby gene, but where a biological connection to SLE was not obvious (Supplementary Table S3, Supplementary Fig. S4).

**Table 2:** De novo SNVs with potential to increase the risk of SLE due to their location in promoters overlapping DNAse 1 hypersensitive sites.

Gene	SNV position in genome^a^	No of TFBS in promotor, expression in blood cells^b,c,d^	FDR *p* value for differential expression^e^	Genes with functional roles potentially relevant to SLE^f^
*AIDA*	NC_000001.10: g.222886101A > G*^,m^	29 TFBS High expression	2.28E–12	Inhibitor of JNK activation giving rise to organ damage in SLE.
*CLOCK*	NC_000004.11: g.56412867C > T	7 TFBS High expression	4.28E–13	Daily variation of NF-κB immune response.
*SQSTM1*	NC_000005.9: g.179248888T > A*^,m^	13 TFBS High expression	6.96E–17	Autophagy receptor involved in TLR7 and NOTCH1 signaling.
*HIST1H1B*	NC_000006.11: g.27835216G > A*	19 TFBS Over-expression	0.000478	Histone protein recognized by SLE autoantibodies.
*DOCK8*	NC_000009.11: g.215166C > T^g^	29 TFBS Over-expression	1.56E–11	Affects survival of B cells. Rare SLE-associated SNPs in gene.
*STOM*	NC_000009.11: g.124132499C > A	16 TFBS Over-expression	0.0000115	Marker for distinguishing CD4 + Th1 from Th2 cells. Gene with Inflammatory role.
*IFIT1*	NC_000010.10: g.91152143A > C*	5 TFBS High expression	4.41E–22	Overexpressed in SLE patients.
*CAND1*	NC_000012.11: g.67663050G > A*	25 TFBS Expressed	4.7E–09	Reduces regulatory T cell functions.
*SRP54*	NC_000014.8: g.35452295G > A*	20 TFBS Over-expression	8.35E–10	Increased reactivity of SRP54 autoantigens against SLE. Associated with increased risk of stroke in SLE.
*DICER*	NC_000014.8: g.95623820G > A	20 TFBS Low expression (all tissues)	0.00675	Reduced in autoimmune MRL/lpr mouse model.
*RNF126*	NC_000019.9: g.661757C > T	2 TFBS Over-expression	7.96E–08	Class I MHC-mediated antigen presentation.
*BAX*	NC_000019.9: 49458424G > A*	7 TFBS Over-expression	1.71E–12	BAX is down-regulated in bone marrow cells from SLE patients and Bcl-2 to BAX ratio elevated in active SLE.

*Validated by Sanger sequencing.

*JNK* c-Jun N-terminal kinase.

^a^Six of the positions are included in the dbSNP, but none has a minor allele frequency >0.0004.

^b^All variants are annotated to promoters and DNase I hypersensitive sites.

^c^TFBS = Number of transcription factor binding sites in B cells.

^d^Expression levels refer to the highest expression in any blood cell type compared with other tissue types, where over-expression means blood tissue specific high expression and high expression means high expression in several tissues including blood tissues.

^e^Differential expression calculated using published gene expression omnibus (GEO) data from two studies on SLE patients and healthy controls [25, 26].

^f^References are found in Supplementary Table S2.

^g^Male patient.

In addition to de novo SNVs in gene promoters, three nonsynonymous de novo SNVs located in coding regions of the *MAZ, LTB4R2,* and *ISX* genes were identified. None of these genes had any nonsynonymous de novo SNVs in the controls. These nsSNVs affect zinc binding to the protein, truncate the protein or affect the DNA-binding, respectively, and thereby most likely affect the function of these proteins (Table 3, Supplementary Table S4). *LTB4R2* is a receptor for the inflammation mediator leukotriene B4 that have been found to be greatly elevated in serum from SLE patients compared with healthy controls [23]. The other two genes play a role in the immune system, but have not previously been implicated in the etiology of SLE.

**Table 3:** De novo nonsynonymous SNVs with potential to affect SLE risk.

Gene	Position (amino acid change, transcript annotation, and genomic coordinate)	Potential effect on protein	Blood cell type with reported highest expression	FDR *p* value for differential expression^a^	Function potentially relevant to SLE^b^
*MAZ*	P56270-1: p.(Cys368Gly) NM_002383.3: c.1276T > G NC_000016.9: g.29819609T > G*	Disrupts zinc binding in C2H2-type zinc finger.	PBMC, NK	9.40E–07	Regulates inflammation-responsive genes.
*LTB4R2*	Q9NPC1-1: p.(Gln169*) NM_019839.4: c.620C > T NC_000014.8: g.24780375C > T	Truncated protein (4 transmembrane helices missing).	PBMC	0.301	Reduced mRNA expression in CD4( + ) T cells in asthma patients.
*ISX*	Q2M1V0-1: p.(Arg138Gln) NM_001303508.1: c.1076G > A NC_000022.10: g.35480407G > A*	Disrupts DNA binding in the major DNA groove.	–	0.929	Regulates Vitamin A which is reduced in SLE patients.

*Validated by Sanger sequencing.

^a^Differential expression calculated using published gene expression omnibus (GEO) data from two studies on SLE patients and healthy controls [25, 26].

^b^References are found in Supplementary Table S4.

### De novo insertion-deletions and structural variants in SLE patients

De novo INDELs are more infrequent than SNVs, but could have larger effects on gene regulation and protein function than SNVs, for example by introducing frameshifts. The low frequency of INDELs makes this study underpowered for detection of INDEL enrichment in regions or genes that are relevant for SLE. Despite this limitation we detected 5 de novo INDELs that were located in the vicinity of the *TPR, CFLAR, RACGAP1, ARID3B, GSDMD* genes that are relevant for SLE (Supplementary Table S5). Structural variants have even greater potential than SNVs and INDELs to affect the function of proteins due to their larger size. However, de novo SVs are infrequent in the human genome, with an expected frequency based on the data from the Icelandic population of ~13 large de novo SVs in our entire trio dataset [24]. Initially, we identified 97 candidate de novo SVs in the SLE patients. Based on their genomic annotations and manual inspection of the sequence reads in the integrated genome browser (IGV) 10 high confidence variants remained for further investigation. Of these 10 SVs, three SVs were close to genes or in genes that could contribute to increased risk of SLE, see Table 4 and Supplementary Table S6. Two of these three are involved in IFN-kappaB signaling.

**Table 4:** De novo structural variants with potential to affect SLE.

Gene	Structural variant position	Length (bp)	Genomic effect	Gene function potentially relevant to SLE^a^
*RBM10*	NC_000023.10:g.47017419_47037305del	19,887	Deletion of exons 3–7	Regulates the activity of NF-κB-responsive promoters and consequently inflammation development.
*SMARCA2*	NC_000009.11:g.2194227_2197614del	3388	Deletion 603 bases downstream	Downregulated in SLE patients with the MECP2 risk haplotype. The patient has the protective haplotype and high expression of SMARCA2.
*PPARA*	NC_000022.10:g.46625870_46626103del	234	Deletion 80 kb from TSS in intron 6	Regulates differentiation of T cells and contributes to the regulating of the activity of NF-KappaB.

*TSS* transcription start site.

^a^References are found in Supplementary Table S6.

## Discussion

As DNMs are not inherited, they do not explain any of the “missing heritability” in SLE, but they may contribute to the genetic risk of obtaining SLE in individual patients. We identified 12 de novo SNVs that may confer risk of SLE based on their position in active gene promoters and based on literature searches of the functions of the genes carrying de novo SNVs. Several of the candidate genes with de novo SNVs do not have a previously known connection to SLE, and thus they represent potential novel risk genes for SLE. However, as our strategy to identify risk genes was based on their known functions related to SLE, there should be a number of additional de novo SNVs than those high-lighted here with potential to increase the genetic risk of SLE.

The enrichment of de novo SNVs in gene promoters in SLE patients compared with the data from the healthy individuals in the Icelandic population amounts to 26 de novo SNVs more than expected by chance, which indicates that de novo SNVs in promoters are a common factor for increasing the genetic risk of SLE. The finding of de novo SNV enrichment in promoters would benefit from replication in another SLE or autoimmune patient cohort. The 12 most plausible de novo SNVs located close genes that are relevant for SLE identified here are distributed across 11 patients. As there are no signs of clustering of de novo SNVs in promoters within individual SLE patients, disease affecting de novo SNVs can be expected to occur in about one-third of SLE patients.

In our study, we found 21 de novo SNV more than expected by chance that are located in promoters overlapping DHS in the SLE patients compared with controls. Assuming a uniform probability distribution of the de novo SNVs between samples, there are 18 SLE patients that have at least one potentially risk-contributing de novo SNV, which correspond to a frequency of 0.26 in our set of SLE patients and an odds ratio of 1.81 compared with controls. These numbers are comparable to for example the *IRF5* locus which is one of the most strongly associated loci in SLE [3, 25]. This comparison suggests that de novo SNVs may contribute significantly to the total burden of risk for SLE caused by genetic variation.

The control data with de novo SNVs in the Islandic population was a valuable asset for our study. The data are technically similar to our data in terms of sequencing coverage and methods used for read mapping and SNV calling. However, the Islandic project used three different sequencing library preparation methods. In order to make the comparison of the de novo SNV data in healthy individuals from Iceland with our data from Swedish patients with SLE as precise as possible, we filtered out de novo SNVs in overlapping regions with high GC-content in both data sets. We found that GC-content filtering had only a marginal effect on the enrichment and significance of de novo SNVs, even in promoter regions which are GC-rich.

Several SLE associated INDELs and SVs have previously been reported in SLE, which are exemplified by a 5 bp INDEL close to *IRF5* [26], copy number variation of *FCGR3A* and *FCGR3B* [27] in synergy with copy number variation of *ADAM3A* [28], and copy number variation of *C4* [29], as well as population specific CNVs in *TLR7, DEFB4, RABGAP1L, and HLA-DRB5* [28]. The CNVs that span protein-coding regions have a dose effect on the expressed proteins that correlates with the copy number. The heterozygous deletion of several exons in *RBM10* detected in this study will also have a dose effect as only one of the chromosomes will produce a functional protein. Rbm10 deficiency suppresses NF-κB-mediated responses in vivo in mouse models and in vitro [30], which supports role for the de novo SVs in increasing SLE risk as NF-κB-activity in T-cells from SLE patients has been shown to be greatly decreased [31].

The fact that de novo SNVs are enriched to promoter regions and not in protein-coding exons highlights the importance of using WGS for identification of de novo variants. However, in a larger patient cohort it is probable that also nonsynonymous de novo SNVs would be statistically significantly enriched. In this study, we found only three potentially SLE risk contributing de novo SNVs that were located in protein coding regions, which is in line with a previous study that identified a small number of de novo variants with large potential to affect the protein function by whole-exome sequencing of SLE trio families [16]. Although the missense de novo SNVs are uncommon they might confer a large risk of SLE for the individual patient and therefore important for understanding the genetic risk underlying SLE.

SLE is considered as a model for systemic autoimmune diseases because the autoimmune process in SLE may affect most organs of the human body. Thus the finding of de novo variants in SLE could also be highly relevant for other systemic autoimmune diseases.

## Methods

### DNA samples from trio families

DNA was extracted from peripheral whole blood of 71 patients affected by SLE and their biological parents, who visited the rheumatology clinics in Uppsala (*n* = 22), Karolinska Hospital (Stockholm) (*n* = 30), Lund (*n* = 15), and Linköping (*n* = 4). All patients were examined by a rheumatologist and their medical records were reviewed. SLE patients and their parents provided informed consent to participate in the study, and the study was approved by the regional ethics committees. Of the patients 85% were female, and on average 24 years old (range 9–46) at onset of SLE. Fourteen of the patients were under 18 years old at SLE onset and could therefore be classified as having childhood-onset SLE. The patients fulfilled at least four American College of Rheumatology (ACR) 1982 criteria for SLE [32], with the exception of five patients who displayed three ACR criteria together with a clinical diagnosis of SLE. For further clinical data, see Supplementary Table S7. All patents included in the study were of European descent.

### Whole-genome sequencing and sequence alignment

Sequencing libraries were prepared from 1 μg DNA using the TruSeq PCR-free DNA sample preparation kit (Illumina Inc.) targeting an insert size of 350 bp. 150 bp paired-end WGS was performed to >30 coverage using an Illumina HiSeqX sequencer with v2.5 chemistry.

The sequences were aligned with BWA-MEM [33] version 0.7.12 using default parameters with the addition of the –M and –R flag, and using the b37 human reference genome from the GATK file bundle version 2.8. The raw alignments where then flagged for duplication, realigned, and recalibrated using GATK version 3.3.0 [34]. Metrics from the WGS after mapping and variant calling are shown in Supplementary Table S8.

### Calling of single nucleotide variants and insertion-deletions

SNVs and INDELs in the WGS data were called jointly for all samples using GATK version 3.5.0 following the GATK best practice protocol. In the variant recalibration step we used positive training data from the HapMap project (with phred quality score prior likelihood of Q15, which is equal to 97% likelihood that the genotype is correct) and the 1000 Genomes Omni 2.5 M genotyping data (prior Q12, 94% likelihood for correct genotypes) as well as in-house genotype data on the samples from the Infinium OmniExpressExome-8 v1.3 SNP chip (Illumina) with 958,497 SNP markers (prior Q20, 99% likelihood). As additional training data we used the 1000 Genomes high confidence calls (prior Q10, 90% likelihood) and for annotation and statistics we used the dbSNP version 138 (prior Q2, 37% likelihood). All data files, except the in-house OmniExpressExome SNP genotype data, were obtained from the GATK file bundle version 2.8. Variants were marked as PASS if the variant quality score log-odds were higher than that in the 99th percentile in the training data for both SNVs and INDELs. The variants were then further refined by calculating genotype posterior likelihoods using the data from parent-offspring trios in GATK. Low quality variants were discarded if the genotype posterior had a score <Q20.

### Calling and filtering of de novo candidate single nucleotide variants and insertion-deletions

De novo SNV and INDEL candidates were called using two programs: GATK version 3.5.0 and Triodenovo [35] version 0.04. By GATK de novo variants were called with the VariantAnnotator using –annotation PossibleDeNovo and Triodenovo was run with default parameters. The program DNMFilter [36] v0.1.1 was used to discern true de novo SNVs from false ones. DNMFilter assigns a score to each de novo SNV candidate that correlates with the probability that the candidate is a true de novo SNV. The score threshold was set to 0.5. The model used by the program is based on the training data supplied in the program package using default features extracted from 264 trio samples. The raw de novo INDEL calls were filtered and annotated as described in the Supplementary “Methods and Results”.

### Validation of de novo single nucleotide variants and insertion-deletions

Primers for PCR were designed using Primer 3 v4.0.0 [37], and the specificity of the primers was confirmed using the BLAST-like alignment tool [38] and in silico PCR. Whole-genome amplification of 50–100 ng of DNA was performed using the REPLI-g Mini Kit (Qiagen), followed by specific PCRs using reagents from the Phusion Hot Start II kit. After amplification the PCR products with correct size were cleaned using Exo/SAP reagents (GE healthcare), and the concentration and size of the PCR products were determined using Qubit HS (Invitrogen). Twenty ng of each PCR product were mixed with 4 pmol of primer and subjected to Sanger sequencing on a Genetic Analyser (Life Technologies). The sequencing results for the SNVs were read using the program Sequence Scanner v2.0, whereas the INDELs were analysed using Chromas Lite v2.3.

To assess the performance of the de novo variant calling pipeline, 110 SNVs and 83 INDELs were randomly selected for validation by Sanger sequencing in all family members. For the SNVs, the primer construction and the sequencing was successful for 99 of the primer pairs, out of which 98 were confirmed to be de novo variants, which corresponds to an FDR of 1%. Sequencing of the INDELs returned results for 71 of the primer pairs, out of which 56 were confirmed to be de novo, which corresponds to an FDR of 21%. The high accuracy of our data and the pipeline for calling de novo SNVs is demonstrated by an allele balance between 30 and 70% for 98% of the called the de novo SNVs after filtering [17, 39]. Thus the called de novo SNVs contained very few mosaic variants and few variants were called due to sequencing errors. Notably, before filtering the majority of the variant alleles were outside of the 30–70% range, see Supplementary Fig. S5. In addition to the randomly selected variants, eight of the variants that could potentially confer risk for SLE (Table 2) were confirmed to be de novo and none of them failed validation. The single potential disease related INDEL tested was also confirmed to be a de novo variant.

### Calling and filtering de novo structural variants

Germline SVs in the form of INDELs larger than 10 base pairs were called by Manta-1.0.3 [40] in samples from parents and SLE patients using default parameters and additionally by FermiKit-0.13 [41] in the patients using the parameter –s3g for genome size and 150 for read-length.

Because Manta and FermiKit do not inherently support calling of de novo variants we developed our own calling and filtering procedure for de novo SVs based on germline variant calls, with a focus on high specificity for variant calling. To call de novo variants we first selected variants exceeding 10 base pairs in the SLE patients that were detected by both FermiKit and Manta using a reciprocal overlap of 50%. FermiKit and Manta complement each other as they use different approaches to call SVs. Using only overlapping calls should therefore greatly reduce the number of false positives. Structural variants of the same type that remained undetected, using a reciprocal overlap of 50%, in any of the trio family samples were kept. The overlap was calculated using both the actual called SVs and the candidate variants generated by the programs. The remaining SVs were annotated similarly as for INDEL filtering, using annotations from blacklisted regions by ENCODE, mappability scores, segmental duplications, and RepeatMasker. In addition, structural variant regions were removed if the coverage in the region flanking the variant followed a similar pattern between an SLE patient and any of the two parents. The presence of the de novo SVs was also confirmed manually in the integrated genome viewer (IGV).

### Functional annotation of de novo single nucleotide variants and insertion-deletions

The variants were annotated functionally using Annovar version 2016.05.11 [42] with the annotations data described below supplied to Annovar via the. Chromatin states were annotated according to ChromHMM [18] predictions based on the B-lymphocyte cell line GM12878. Relative gene positions were obtained using RefSeq [43]. TFBS were annotated using the chromatin-immunoprecipitaion sequencing (ChIP-seq)-based UCSC genome browser track wgEncodeRegTfbsClustered from ENCODE. DNase I hypersensitive site (DHS) annotations were based on the UCSC genome browser track wgEncodeOpenChromDnaseGm12878Pk based on the GM12878 cell line from ENCODE. The effects of de novo nsSNVs on the functions of the encoded proteins were determined using positional annotations from the UniProtKB [44], 3-dimensional protein structures in the protein data bank (PDB) [45] or structural models in the Protein Model Portal [46]. Gene expression data of nearby genes was obtained from GeneCards, collected from the Human Integrated Protein Expression Database (www.genecards.org, [47, 48]). We also used the USCS genome browser to manually inspected de novo SNVs in promoter regions in to find de novo SNVs with large potential to affect transcription factor binding due to their positions in relation to TFBS, histone marks, and open chromatin in B-lymphocytes [20].

### Parent of origin and age at conception

The parent of origin of de novo SNVs in the offspring was determined in the genomes of 32 trio family members for those variants that had an additional informative SNV within the same sequencing read (*n* = 342 de novo SNVs). At least two reads spanning both the additional SNV and the de novo SNV supporting the same parent was required to assign the parental origin of a de novo SNV.

The number of expected de novo SNVs in the offspring is affected by the age at conception of the father and to a lesser extent the age of the mother [8]. The correlation and *p* value of the correlation between age of the father and the child affected with SLE was calculated using linear regression in R.

### De novo single nucleotide variants in healthy controls

A set of 97,942 de novo SNV and over 30 million SNVs found in 1548 trios from a study in Iceland by deCODE Genetics was downloaded from the European Variation Archive (www.ebi.ac.uk/eva/), study accession number PRJEB15197, to be used as healthy control data in our study [8]. The SNVs in the Icelandic data were then annotated exactly as the de novo SNVs detected in the Swedish SLE trios. For the enrichment analysis we also filtered out de novo SNVs in overlapping regions with high GC-content in both datasets. The SNVs filtered were located in 100 bp regions with at least 75% GC content or located in any of the 1000 “difficult promoters” defined by Ross et al. [49].

Among the Icelandic de novo SNVs, 87 were randomly selected to match the number of SNVs found in the Swedish SLE patients. The de novo SNVs in Iceland were visually inspected in the UCSC genome browser in an identical fashion as the de novo SNVs in the SLE patients to determine a subset of de novo SNVs most likely to affect the promoter. Enrichment *p* values were calculated using the Pearson’s chi-squared test with Yates’ correction.

### Calculation of odds ratios and significance in selected annotated regions

Odds ratios between de novo SNVs found in our SLE cohort and the controls were calculated for selected chromatin states determined by ChIP-seq annotations in gene flanking regions and intergenic regions, DHS, number of TFBS, and combinations of these regions. The significance of the enrichment was then calculated using Pearson’s chi-squared test.

### Differential gene expression data sets

We used two publicly available gene expression datasets deposited in GEO [50] to calculate FDR corrected *p* values for differential expression between SLE patients and controls using the online service GEO2R (https://www.ncbi.nlm.nih.gov/geo/geo2r/). One of the studies (GEO accession number: GSE45291) contains expression array data for 54715 probes from 292 SLE patients and 20 controls [21]. The other study (GEO accession number: GSE65391) is a longitudinal study of pediatric SLE patients where the sample taken at the first visit to the clinic was used to calculate differential gene expression for 43,798 probes between 134 SLE patients and 36 controls [22]. The lowest probe *p* value for each gene from the two studies was used in our study.

## Supplementary information

Supplemental Figures S1,S2,S5 and Tables S1,S3,S5,S8

Supplemental Figure S3

Supplemental Figure S4

Supplemental Table S2

Supplemental Table S4

Supplemental Table S6

Supplemental Table S7

Supplemental Material and Results

## Data Availability

De novo SNVs and INDELs presented in this paper have been submitted to dbSNP (https://www.ncbi.nlm.nih.gov/projects/SNP/) under the submitter ID MOLMED_UU and Submitter batch ID Denovo_variants_1.0 with the submission SNP IDs of ss2137544038 and ss3798736965 to ss3798737055.

## References

[1] Stahl-Hallengren C, Jonsen A, Nived O, Sturfelt G (2000). Incidence studies of systemic lupus erythematosus in Southern Sweden: increasing age, decreasing frequency of renal manifestations and good prognosis. J Rheumatol..

[2] Rees F, Doherty M, Grainge MJ, Lanyon P, Zhang W (2017). The worldwide incidence and prevalence of systemic lupus erythematosus: a systematic review of epidemiological studies. Rheumatol (Oxf).

[3] Langefeld CD, Ainsworth HC, Cunninghame Graham DS, Kelly JA, Comeau ME, Marion MC (2017). Transancestral mapping and genetic load in systemic lupus erythematosus. Nat Commun.

[4] Chen L, Morris DL, Vyse TJ (2017). Genetic advances in systemic lupus erythematosus: an update. Curr Opin Rheumatol.

[5] Morris DL, Sheng Y, Zhang Y, Wang YF, Zhu Z, Tombleson P (2016). Genome-wide association meta-analysis in Chinese and European individuals identifies ten new loci associated with systemic lupus erythematosus. Nat Genet..

[6] Costa-Reis P, Sullivan KE (2017). Monogenic lupus: it’s all new!. Curr Opin Immunol.

[7] Almlof JC, Nystedt S, Leonard D, Eloranta ML, Grosso G, Sjowall C (2019). Whole-genome sequencing identifies complex contributions to genetic risk by variants in genes causing monogenic systemic lupus erythematosus. Hum Genet..

[8] Jonsson H, Sulem P, Kehr B, Kristmundsdottir S, Zink F, Hjartarson E (2017). Parental influence on human germline de novo mutations in 1,548 trios from Iceland. Nature..

[9] Veltman JA, Brunner HG (2012). De novo mutations in human genetic disease. Nat Rev Genet.

[10] Zaidi S, Choi M, Wakimoto H, Ma L, Jiang J, Overton JD (2013). De novo mutations in histone-modifying genes in congenital heart disease. Nature..

[11] Yang Y, Muzny DM, Xia F, Niu Z, Person R, Ding Y (2014). Molecular findings among patients referred for clinical whole-exome sequencing. JAMA..

[12] Deciphering Developmental Disorders S. (2017). Prevalence and architecture of de novo mutations in developmental disorders. Nature..

[13] Neale BM, Kou Y, Liu L, Ma’ayan A, Samocha KE, Sabo A (2012). Patterns and rates of exonic de novo mutations in autism spectrum disorders. Nature..

[14] Gonzalez KD, Buzin CH, Noltner KA, Gu D, Li W, Malkin D (2009). High frequency of de novo mutations in Li-Fraumeni syndrome. J Med Genet.

[15] Duncan CJA, Dinnigan E, Theobald R, Grainger A, Skelton AJ, Hussain R (2018). Early-onset autoimmune disease due to a heterozygous loss-of-function mutation in TNFAIP3 (A20). Ann Rheum Dis.

[16] Pullabhatla V, Roberts AL, Lewis MJ, Mauro D, Morris DL, Odhams CA (2018). De novo mutations implicate novel genes in systemic lupus erythematosus. Hum Mol Genet.

[17] Besenbacher S, Liu S, Izarzugaza JM, Grove J, Belling K, Bork-Jensen J (2015). Novel variation and de novo mutation rates in population-wide de novo assembled Danish trios. Nat Commun.

[18] Ernst J, Kellis M (2012). ChromHMM: automating chromatin-state discovery and characterization. Nat Methods..

[19] Karrar S, Cunninghame Graham DS (2018). Abnormal B cell development in systemic lupus erythematosus: what the genetics tell us. Arthritis Rheumatol..

[20] Kent WJ, Sugnet CW, Furey TS, Roskin KM, Pringle TH, Zahler AM (2002). The human genome browser at UCSC. Genome Res.

[21] Petri M, Fu W, Ranger A, Allaire N, Cullen P, Magder LS (2019). Association between changes in gene signatures expression and disease activity among patients with systemic lupus erythematosus. BMC Med Genomics.

[22] Banchereau R, Hong S, Cantarel B, Baldwin N, Baisch J, Edens M (2016). Personalized immunomonitoring uncovers molecular networks that stratify lupus patients. Cell..

[23] Wu T, Xie C, Han J, Ye Y, Weiel J, Li Q (2012). Metabolic disturbances associated with systemic lupus erythematosus. PLoS One..

[24] Brandler WM, Antaki D, Gujral M, Noor A, Rosanio G, Chapman TR (2016). Frequency and complexity of de novo structural mutation in autism. Am J Hum Genet.

[25] Sigurdsson S, Nordmark G, Goring HH, Lindroos K, Wiman AC, Sturfelt G (2005). Polymorphisms in the tyrosine kinase 2 and interferon regulatory factor 5 genes are associated with systemic lupus erythematosus. Am J Hum Genet.

[26] Sigurdsson S, Goring HH, Kristjansdottir G, Milani L, Nordmark G, Sandling JK (2008). Comprehensive evaluation of the genetic variants of interferon regulatory factor 5 (IRF5) reveals a novel 5 bp length polymorphism as strong risk factor for systemic lupus erythematosus. Hum Mol Genet.

[27] Qi Y, Zhou X, Bu D, Hou P, Lv J, Zhang H (2017). Low copy numbers of FCGR3A and FCGR3B associated with Chinese patients with SLE and AASV. Lupus..

[28] Barbosa FB, Simioni M, Wiezel CEV, Torres FR, Molck MC, Bonilla MM (2018). Copy number variation in the susceptibility to systemic lupus erythematosus. PLoS ONE.

[29] Chen JY, Wu YL, Mok MY, Wu YJ, Lintner KE, Wang CM (2016). Effects of complement C4 gene copy number variations, size dichotomy, and C4A deficiency on genetic risk and clinical presentation of systemic lupus erythematosus in East asian populations. Arthritis Rheumatol..

[30] Atsumi T, Suzuki H, Jiang JJ, Okuyama Y, Nakagawa I, Ota M (2017). Rbm10 regulates inflammation development via alternative splicing of Dnmt3b. Int Immunol.

[31] Wong HK, Kammer GM, Dennis G, Tsokos GC (1999). Abnormal NF-kappa B activity in T lymphocytes from patients with systemic lupus erythematosus is associated with decreased p65-RelA protein expression. J Immunol.

[32] Tan EM, Cohen AS, Fries JF, Masi AT, McShane DJ, Rothfield NF (1982). The 1982 revised criteria for the classification of systemic lupus erythematosus. Arthritis Rheum..

[33] Li H, Durbin R (2009). Fast and accurate short read alignment with Burrows-Wheeler transform. Bioinformatics..

[34] McKenna A, Hanna M, Banks E, Sivachenko A, Cibulskis K, Kernytsky A (2010). The Genome Analysis Toolkit: a MapReduce framework for analyzing next-generation DNA sequencing data. Genome Res..

[35] Wei Q, Zhan X, Zhong X, Liu Y, Han Y, Chen W (2015). A Bayesian framework for de novo mutation calling in parents-offspring trios. Bioinformatics..

[36] Liu Y, Li B, Tan R, Zhu X, Wang Y (2014). A gradient-boosting approach for filtering de novo mutations in parent-offspring trios. Bioinformatics..

[37] Untergasser A, Cutcutache I, Koressaar T, Ye J, Faircloth BC, Remm M (2012). Primer3–new capabilities and interfaces. Nucleic Acids Res.

[38] Kent WJ (2002). BLAT–the BLAST-like alignment tool. Genome Res..

[39] Kosmicki JA, Samocha KE, Howrigan DP, Sanders SJ, Slowikowski K, Lek M (2017). Refining the role of de novo protein-truncating variants in neurodevelopmental disorders by using population reference samples. Nat Genet..

[40] Chen X, Schulz-Trieglaff O, Shaw R, Barnes B, Schlesinger F, Kallberg M (2016). Manta: rapid detection of structural variants and indels for germline and cancer sequencing applications. Bioinformatics..

[41] Li H (2015). FermiKit: assembly-based variant calling for Illumina resequencing data. Bioinformatics..

[42] Wang K, Li M, Hakonarson H (2010). ANNOVAR: functional annotation of genetic variants from high-throughput sequencing data. Nucleic Acids Res.

[43] Pruitt KD, Tatusova T, Maglott DR (2007). NCBI reference sequences (RefSeq): a curated non-redundant sequence database of genomes, transcripts and proteins. Nucleic Acids Res.

[44] The UniProt C: UniProt. (2017). the universal protein knowledgebase. Nucleic Acids Res.

[45] Berman HM, Westbrook J, Feng Z, Gilliland G, Bhat TN, Weissig H (2000). The protein data bank. Nucleic Acids Res.

[46] Haas J, Roth S, Arnold K, Kiefer F, Schmidt T, Bordoli L (2013). The Protein Model Portal–a comprehensive resource for protein structure and model information. J Biol Databases Curation.

[47] Stelzer G, Rosen N, Plaschkes I, Zimmerman S, Twik M, Fishilevich S (2016). The GeneCards suite: from gene data mining to disease genome sequence analyses. Curr Protoc Bioinforma.

[48] Fishilevich S, Zimmerman S, Kohn A, Iny Stein T, Olender T, Kolker E, et al. Genic insights from integrated human proteomics in GeneCards. Database. 2016;2016:baw030. 10.1093/database/baw030 PMC4820835 27048349

[49] Ross MG, Russ C, Costello M, Hollinger A, Lennon NJ, Hegarty R (2013). Characterizing and measuring bias in sequence data. Genome Biol.

[50] Barrett T, Wilhite SE, Ledoux P, Evangelista C, Kim IF, Tomashevsky M (2013). NCBI GEO: archive for functional genomics data sets–update. Nucleic Acids Res.

