# Supplementary material for: Contributions of de novo variants to systemic lupus erythematosus
Source: Eur J Hum Genet. 2020 Jul 28;29(1):184–93. doi: 10.1038/s41431-020-0698-5 (PMC7852530; doi:10.1038/s41431-020-0698-5)
Supplement: Supplementary file 1 — Supplemental Figures S1,S2,S5 and Tables S1,S3,S5,S8 [file 41431_2020_698_MOESM1_ESM.docx]

# Contributions of *de novo* variants to systemic lupus erythematosus

## European Journal of Human Genetics

Jonas Carlsson Almlöf^1*^, Sara Nystedt^1^, Aikaterini Mechtidou^1^, Dag Leonard^5^, Maija-Leena Eloranta^5^, Giorgia Grosso^4^, Christopher Sjöwall^2^, Anders A. Bengtsson^3^, Andreas Jönsen^3^, Iva Gunnarsson^4^, Elisabet Svenungsson^4^, Lars Rönnblom^5^, Johanna K. Sandling^5^, Ann-Christine Syvänen^1^

^1^Department of Medical Sciences, Molecular Medicine and Science for Life Laboratory, Uppsala University, 751 23 Uppsala, Sweden; ^2^Department of Clinical and Experimental Medicine, Rheumatology/Division of Neuro and Inflammation Sciences, Linköping University, 581 83 Linköping, Sweden; ^3^Department of Clinical Sciences, Rheumatology, Lund University, Skåne University Hospital, 222 42 Lund, Sweden; ^4^Department of Medicine, Karolinska Institutet, Rheumatology, Karolinska University Hospital, 171 77 Stockholm, Sweden; ^5^Department of Medical Sciences, Rheumatology and Science for Life Laboratory, Uppsala University, 751 85 Uppsala, Sweden;


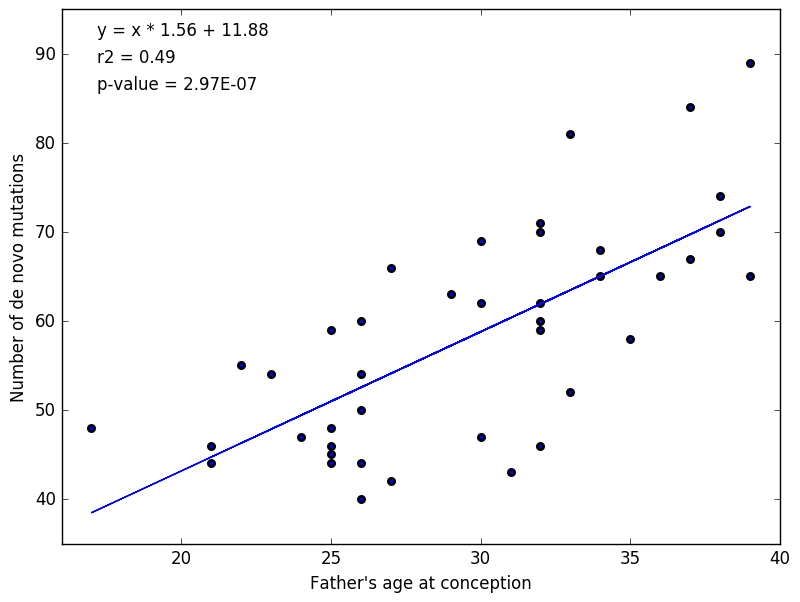


Supplemental Figure S1 Effect of the age of the fathers at conception on the number of *de novo* SNVs in a child. Each dot represents one father-child pair and the solid line shows the linear regression for the data. Data from 32 informative father-child pairs is shown.

Supplemental Figure S2 Enrichment of *de novo* SNVs as a function of the number of annotated transcription binding sites that overlap the positions of *de novo* SNV position.


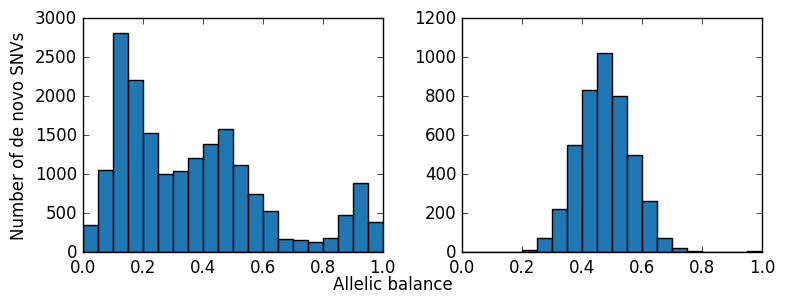


Supplemental Figure S5 Distribution of the allelic balance for *de novo* SNVs before and after removal of *de novo* SNVs with large allelic imbalance using the program DNMFilter. Calling of *de novo* SNVs at high sensitivity comes at a cost of a large proportion of false positives. This was especially true for Triodenovo which called several times more *de novo* SNVs than GATK.

Supplemental Table S1 All d*e novo* SNVs in central parts of predicted B-cell promoters. The *de novo* SNVs where selected due to their positions in relation to transcription factor binding sites, histone marks, and open chromatin in B-cells ([Kent et al., 2002](#_ENREF_3)).

| **Genes** | **Position** | **Variant** | **Differential expression (FDR p-value)^1^** |
| --- | --- | --- | --- |
| *RAB3GAP2* | chr1:220445625 | C>T | 0.00246 |
| *BROX, AIDA* | chr1:222886101 | A>G | 2.28E-12 |
| *FMNL2* | chr2:153193202 | C>T | 0.665 |
| *GALNT3* | chr2:166651380 | A>G | 0.00147 |
| *CLOCK* | chr4:56412867 | C>T | 4.28E-13 |
| *SQSTM1* | chr5:179248888 | T>C | 6.96E-17 |
| *ARRDC3* | chr5:90677756 | T>C | 0.248 |
| *HIST1H1B* | chr6:27835216 | G>A | 0.000478 |
| *LYRM4* | chr6:5260643 | G>A | 1.86E-05 |
| *ZNF292* | chr6:87864999 | C>T | 5.69E-33 |
| *CCDC71L* | chr7:106300401 | T>A | 5.27E-05 |
| *STOM* | chr9:124132499 | C>A | 1.15E-05 |
| *FAM102A* | chr9:130743108 | C>T | 5.54E-07 |
| *C9orf66, DOCK8* | chr9:215166 | C>T | 1.56E-11 |
| *UBAP2* | chr9:34049733 | C>T | 0.0128 |
| *USMG5* | chr10:105155510 | G>T | 1E-07 |
| *POLR3A* | chr10:79788892 | C>G | 0.0233 |
| *IFIT1* | chr10:91152143 | A>C | 4.41E-22 |
| *MTMR2* | chr11:95657216 | C>G | 0.33 |
| *CAND1* | chr12:67663050 | G>A | 4.7E-09 |
| *GTF2F2* | chr13:45693852 | G>C | 1.06E-08 |
| *CLN5* | chr13:77566039 | G>A | 6.75E-05 |
| *SRP54* | chr14:35452295 | G>A | 8.35E-10 |
| *GNPNAT1* | chr14:53258198 | C>A | 0.078 |
| *DICER1* | chr14:95623820 | G>A | 0.00675 |
| *BBS2* | chr16:56554091 | A>G | 1.15E-05 |
| *SDR42E1* | chr16:82045287 | C>A | 0.172 |
| *MED13* | chr17:60142354 | T>C | 2.31E-18 |
| *SEH1L, PTPN2* | chr18:12948527 | C>G | 0.000734 |
| *BAX* | chr19:49458424 | G>A | 1.71E-12 |
| *RNF126* | chr19:661757 | C>T | 7.96E-08 |
| *MYH9* | chr22:36783846 | G>A | 0.623 |
| *MSL3* | chrX:11775971 | C>G | 0.00121 |

^1^Differential expression calculated using published GEO data from two studies on SLE patient and healthy controls ([Banchereau et al., 2016](#_ENREF_1); [Bienkowska et al., 2014](#_ENREF_2)).

Supplemental Table S3 Additional *de novo* SNV candidates located in promoters without obvious biological connection with SLE having the highest potential to affect the expression of the nearby gene

| **Gene** | **Position** | **Relevant annotations^1,2,3^** | **Differential expression (FDR p-value)^4^** | **Protein function** |
| --- | --- | --- | --- | --- |
| *RAB3GAP2* | NC_000001.10:  g.220445625C>T* | 13 TFBS  Highly in specific blood cell type | 0.0000269 | Regulatory subunit of Rab3 GTPase-activating complex involved in regulated exocytosis of neurotransmitters and hormones. |
| *LYRM4* | NC_000006.11:  g.5260643G>A | 10 TFBS  Highly expressed in specific blood cell type | 0.0000186 | Required for nuclear and mitochondrial iron-sulfur protein biosynthesis. |
| *ZNF292* | NC_000006.11:  g.87864999C>T | 17 TFBS  Expressed in specific blood cell type | 5.69E-33 | Transcription factor that functions as a tumor suppressor. |
| *CCDC71L* | NC_000007.13:  g.106300401T>A | 5 TFBS  Expressed in specific blood cell type | 0.0000527 | Unknown |
| *CLN5* | NC_000013.10:  g.77566039G>A | 13 TFBS  Highly expressed in specific blood cell type | 0.0000675 | Expressed in most human tissues. Disease-causing mutations in CLN5 affect intracellular trafficking to lysomes and cause monogenic neuronal ceroid lipofuscinosis (OMIM 256731). |
| *BBS2* | NC_000016.9:  g.56554091A>G | 12 TFBS  Overexpressed in specific blood cell type | 0.0000115 | Part of the BBSome complex which is required for sorting of specific membrane proteins to the primary cilia. |
| *MED13* | NC_000017.10:  g.60142354T>C | 7 TFBS  Expressed in specific blood cell type | 2.31E-18 | Component of the Mediator complex, a coactivator involved in the regulated transcription of nearly all RNA polymerase II-dependent genes. |
| *SEH1L* | NC_000018.9:  g.12948527C>G* | 26 TFBS  Overexpressed in specific blood cell type | 0.000734 | Component of the Nup107-160 subcomplex of the nuclear pore complex (NPC) and is required for NPC assembly, normal kinetochore microtubule attachment, mitotic progression and chromosome segregation. |
| *THEM42* | NC_000003.11:  g.44903329G>A* | 30 TFBS  Expressed in specific blood cell type | 0.642 | Transmembrane protein. |

^1^All variants are annotated to promoters and DNase I hypersensitive sites. ^2^TFBS: Transcription factor binding sites in B-cells. ^3^Expression levels refer to the highest expression of any blood cell type compared to non-blood cell types. ^4^Differential expression calculated using published GEO data from two studies on SLE patient and healthy controls ([Banchereau et al., 2016](#_ENREF_1); [Bienkowska et al., 2014](#_ENREF_2)). *Validated by Sanger sequencing. ^m^Male patient.

Supplemental Table S5 INDELs in the vicinity of potentially SLE relevant genes

| **Gene** | **Position** | **Length (bp)** | **Genomic effect^1^** | **Function potentially relevant to SLE** |
| --- | --- | --- | --- | --- |
| *TPR* | NC_000001.10:g.186345696_186345699delCTTA | 4 | Deletion in promoter affecting 1 TFBS.  TF Hltf are predicted to have small increase in affinity | The majority of patients with anti-Tpr had SLE, SSc or SJS |
| *CFLAR* | NC_000002.11:g.202009412_202009413insC | 1 | Insertion in repetitive intronic region affecting 11 TFBS. TF Zfp423 are predicted to have small increase in affinity | Anti-apoptotic, elevated in SLE patients |
| *RACGAP1* | NC_000012.11:g.50374429delA* | 1 | Deletion in intergenic region 8kb upstream of TSS affecting 6 TFBS | Upregulated sense and downregulated antisense transcripts in SLE |
| *ARID3B* | NC_000015.9:g.74843058_74843059insT | 1 | Insertion in peripheral part of promoter.  TF Hltf are predicted to have small decrease in affinity and RORA_2 and FOXL1 are predicted to have a small increase in affinity | Arid3b Is Critical for B Lymphocyte Development |
| *GSDMD* | NC_000008.10:g.144629404_144629406delAAT | 3 | Intronic (6kb), Enhancer, TFBS = 5, SPIB: increase in affinity | Important in triggering pyroptosis |

*Validated by Sanger sequencing. ^1^Prediction of TF binding affinity changes was performed by sTRAP which was run on the web tool found at http://trap.molgen.mpg.de/cgi-bin/trap_two_seq_form.cgi ([Manke, Heinig, & Vingron, 2010](#_ENREF_4); [Thomas-Chollier et al., 2011](#_ENREF_6)) using the Jaspar motif database ([Mathelier et al., 2016](#_ENREF_5)). A change and small change in affinity equals a change in p-value of at least log(1.5) and log(0.8) respectively. TSS: transcription start site, TF: transcription factor, TFBS: transcription factor binding site, SSc: Systemic Sclerosis, SJS: Stevens-Johnson syndrome

Supplemental Table S8 Statistics of the whole genome sequencing.

| **Statistics** | **Average (Standard deviation)** |
| --- | --- |
| Coverage | 40.4 (7.4) |
| Coverage > 30X | 79.5% (11.3%) |
| Aligned reads | 920M (16M) |
| Percent aligned reads | 99.3% (0.5%) |
| Duplication rate | 12.7% (4.6%) |
| Insert size | 360 (20.4) |
| GC content | 40.8% (0.3%) |
| Ti/Tv ratio | 2.10 |
| SNVs | 13355113 |
| INDELs | 3309183 |
| Insertions | 772838 |
| Deletions | 998747 |
| Missense SNV | 191725 |
| Nonsense SNV | 2754 |
| Silent SNV | 159421 |
| Variants per sample | 3740689 |
| Homozygous variants per sample | 2265531 |
| Heterozygous variants per sample | 1475158 |

# References

Banchereau, R., Hong, S., Cantarel, B., Baldwin, N., Baisch, J., Edens, M., . . . Pascual, V. (2016). Personalized Immunomonitoring Uncovers Molecular Networks that Stratify Lupus Patients. *Cell, 165*(3), 551-565. doi: 10.1016/j.cell.2016.03.008

Bienkowska, J., Allaire, N., Thai, A., Goyal, J., Plavina, T., Nirula, A., . . . Browning, J. L. (2014). Lymphotoxin-LIGHT pathway regulates the interferon signature in rheumatoid arthritis. *PLoS One, 9*(11), e112545. doi: 10.1371/journal.pone.0112545

Kent, W. J., Sugnet, C. W., Furey, T. S., Roskin, K. M., Pringle, T. H., Zahler, A. M., & Haussler, D. (2002). The human genome browser at UCSC. *Genome Res, 12*(6), 996-1006. doi: 10.1101/gr.229102

Manke, T., Heinig, M., & Vingron, M. (2010). Quantifying the effect of sequence variation on regulatory interactions. *Hum Mutat, 31*(4), 477-483. doi: 10.1002/humu.21209

Mathelier, A., Fornes, O., Arenillas, D. J., Chen, C. Y., Denay, G., Lee, J., . . . Wasserman, W. W. (2016). JASPAR 2016: a major expansion and update of the open-access database of transcription factor binding profiles. *Nucleic Acids Res, 44*(D1), D110-115. doi: 10.1093/nar/gkv1176

Thomas-Chollier, M., Hufton, A., Heinig, M., O'Keeffe, S., Masri, N. E., Roider, H. G., . . . Vingron, M. (2011). Transcription factor binding predictions using TRAP for the analysis of ChIP-seq data and regulatory SNPs. *Nat Protoc, 6*(12), 1860-1869. doi: 10.1038/nprot.2011.409
