# Supplementary material for: Contributions of de novo variants to systemic lupus erythematosus
Source: Eur J Hum Genet. 2020 Jul 28;29(1):184–93. doi: 10.1038/s41431-020-0698-5 (PMC7852530; doi:10.1038/s41431-020-0698-5)

# Contributions of *de novo* variants to systemic lupus erythematosus

## Supplemental Figure S3

## European Journal of Human Genetics

Jonas Carlsson Almlöf^1*^, Sara Nystedt^1^, Aikaterini Mechtidou^1^, Dag Leonard^5^, Maija-Leena Eloranta^5^, Giorgia Grosso^4^, Christopher Sjöwall^2^, Anders A. Bengtsson^3^, Andreas Jönsen^3^, Iva Gunnarsson^4^, Elisabet Svenungsson^4^, Lars Rönnblom^5^, Johanna K. Sandling^5^, Ann-Christine Syvänen^1^

^1^Department of Medical Sciences, Molecular Medicine and Science for Life Laboratory, Uppsala University, 751 23 Uppsala, Sweden; ^2^Department of Clinical and Experimental Medicine, Rheumatology/Division of Neuro and Inflammation Sciences, Linköping University, 581 83 Linköping, Sweden; ^3^Department of Clinical Sciences, Rheumatology, Lund University, Skåne University Hospital, 222 42 Lund, Sweden; ^4^Department of Medicine, Karolinska Institutet, Rheumatology, Karolinska University Hospital, 171 77 Stockholm, Sweden; ^5^Department of Medical Sciences, Rheumatology and Science for Life Laboratory, Uppsala University, 751 85 Uppsala, Sweden;

## NC_000001.10:g.222886101A>G in promoter of AIDA


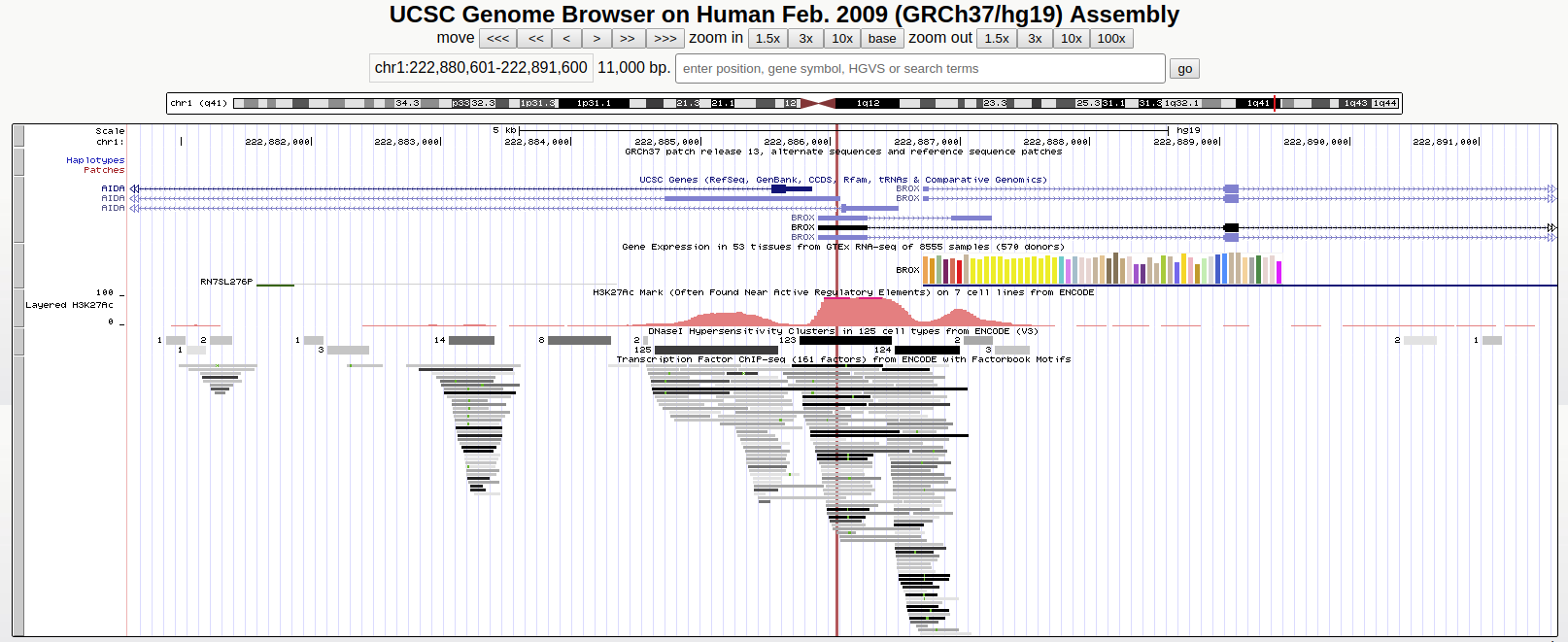


## NC_000009.11:g.215166C>T in promoter of DOCK8


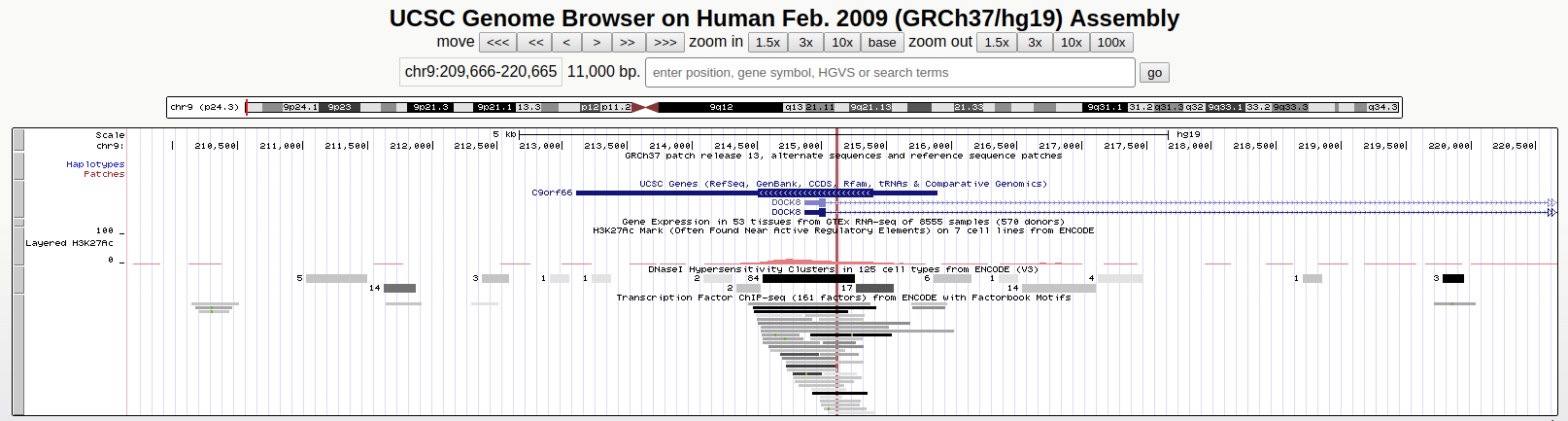


## NC_000014.8:g.35452295G>A in promoter of SRP54


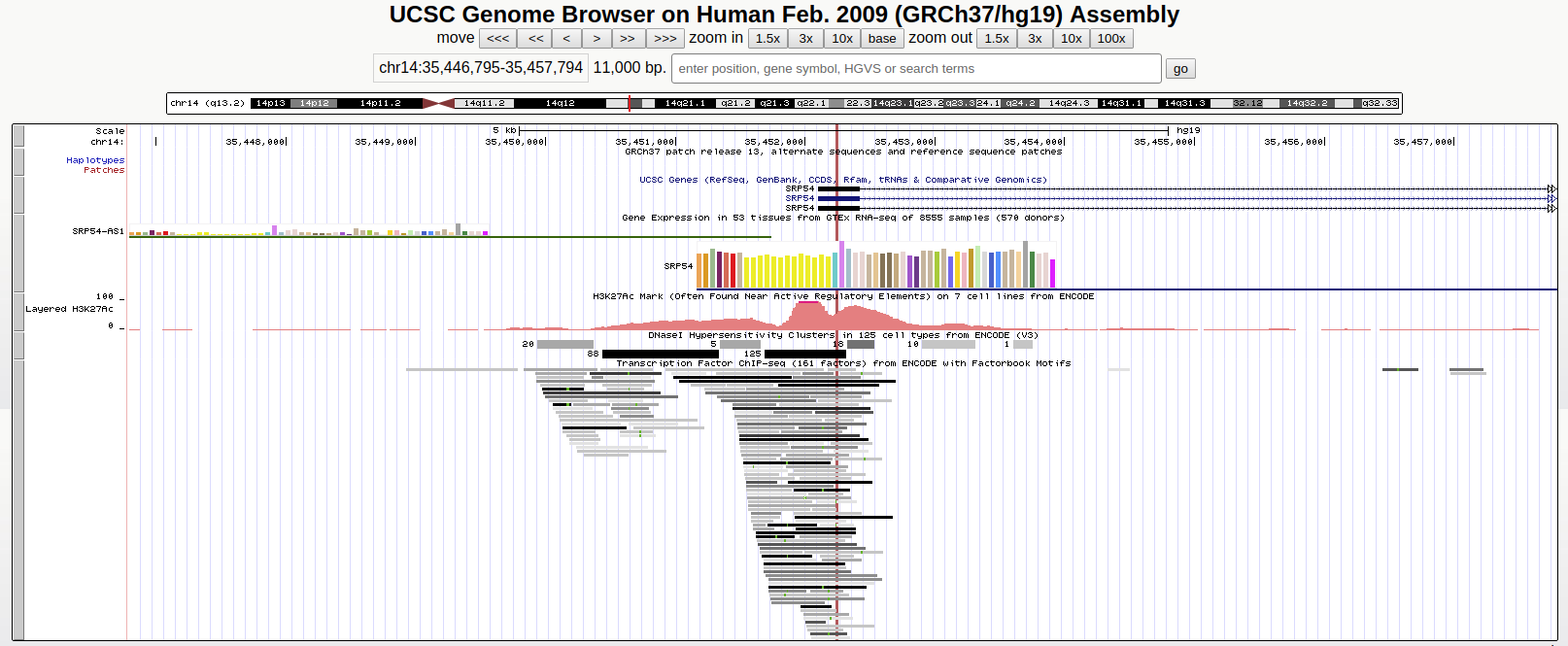


## NC_000012.11:g.67663050G>A in promoter of CAND1


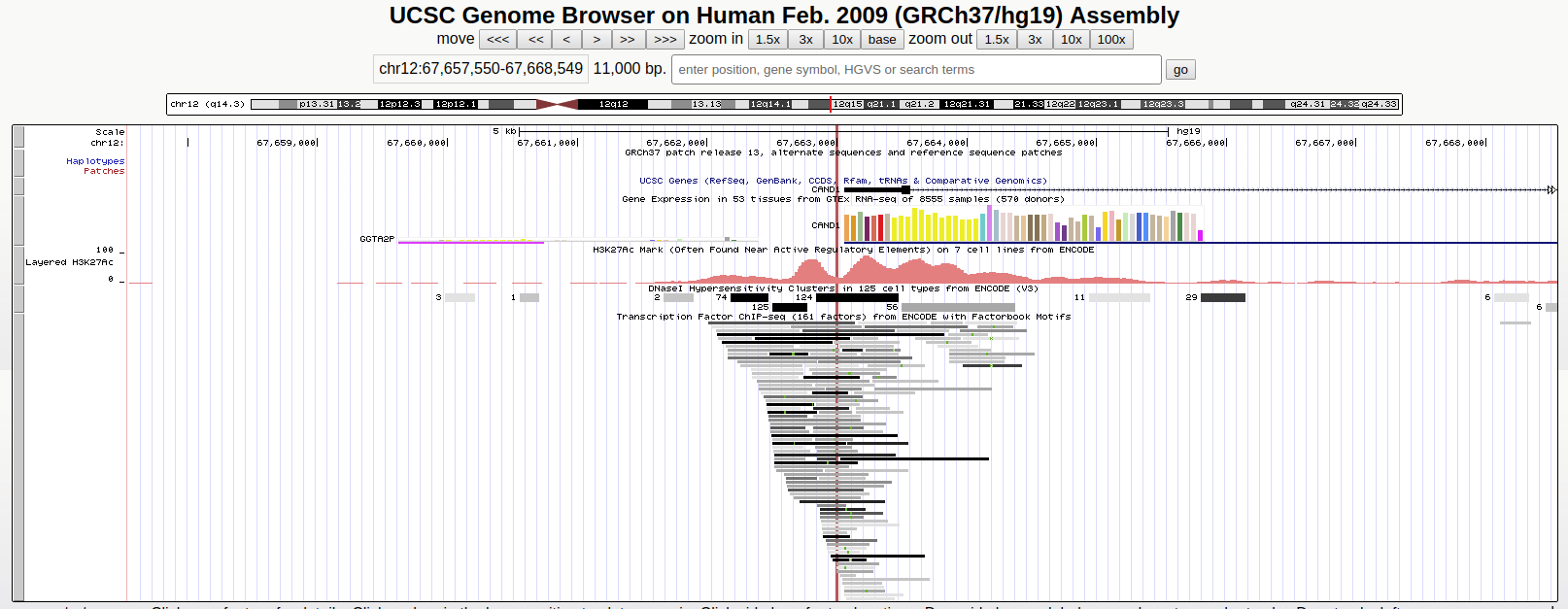


## NC_000006.11:g.27835216G>A in promoter of HIST1H1B


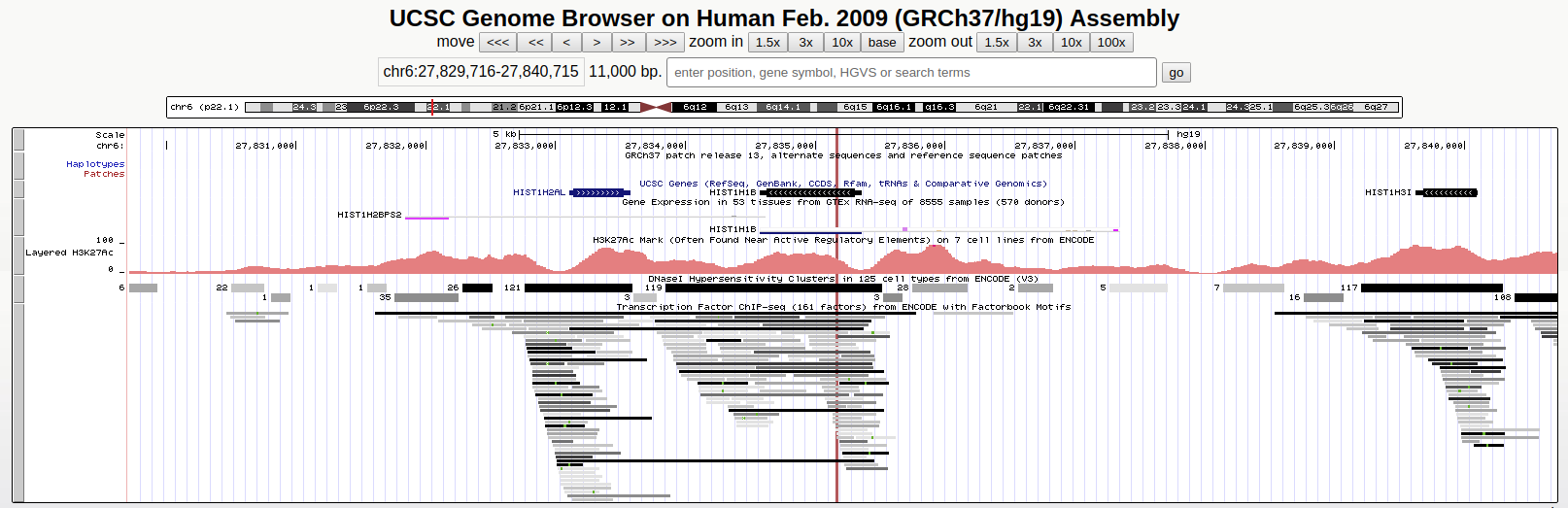


## NC_000009.11:g.124132499C>A in promoter of STOM


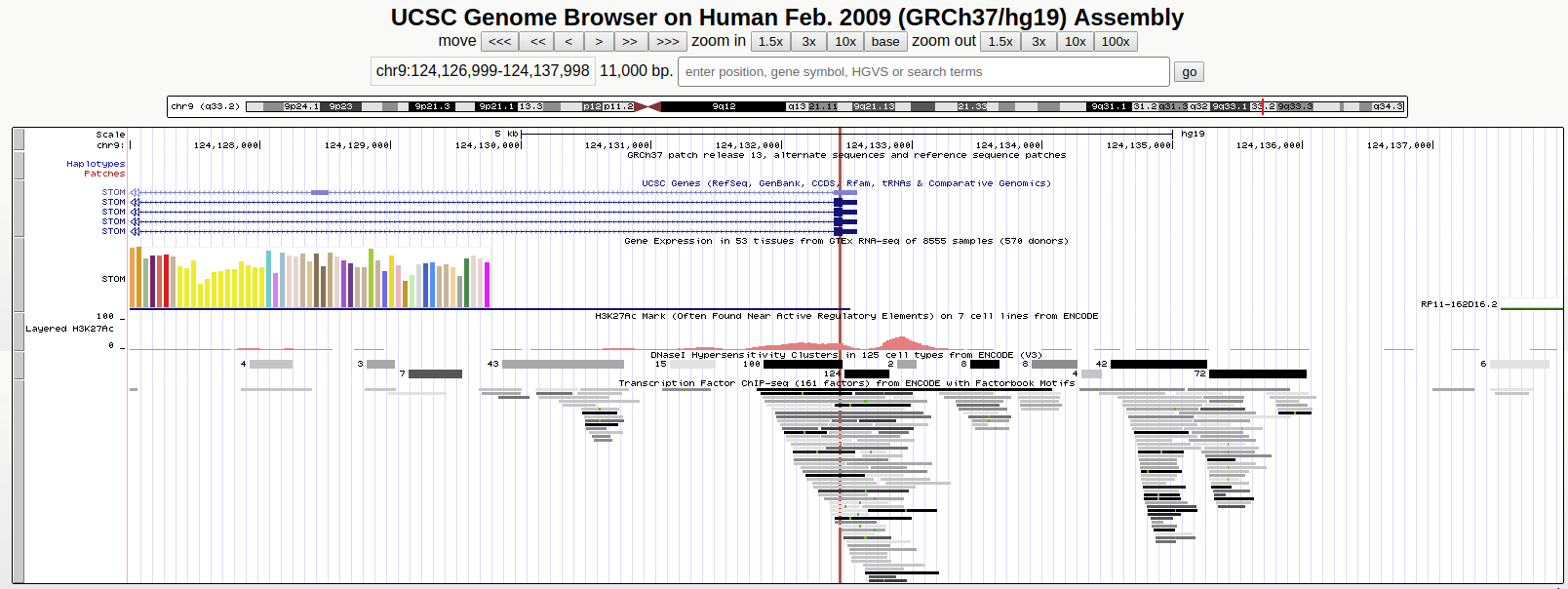


## NC_000019.9:49458424G>A in promoter of BAX


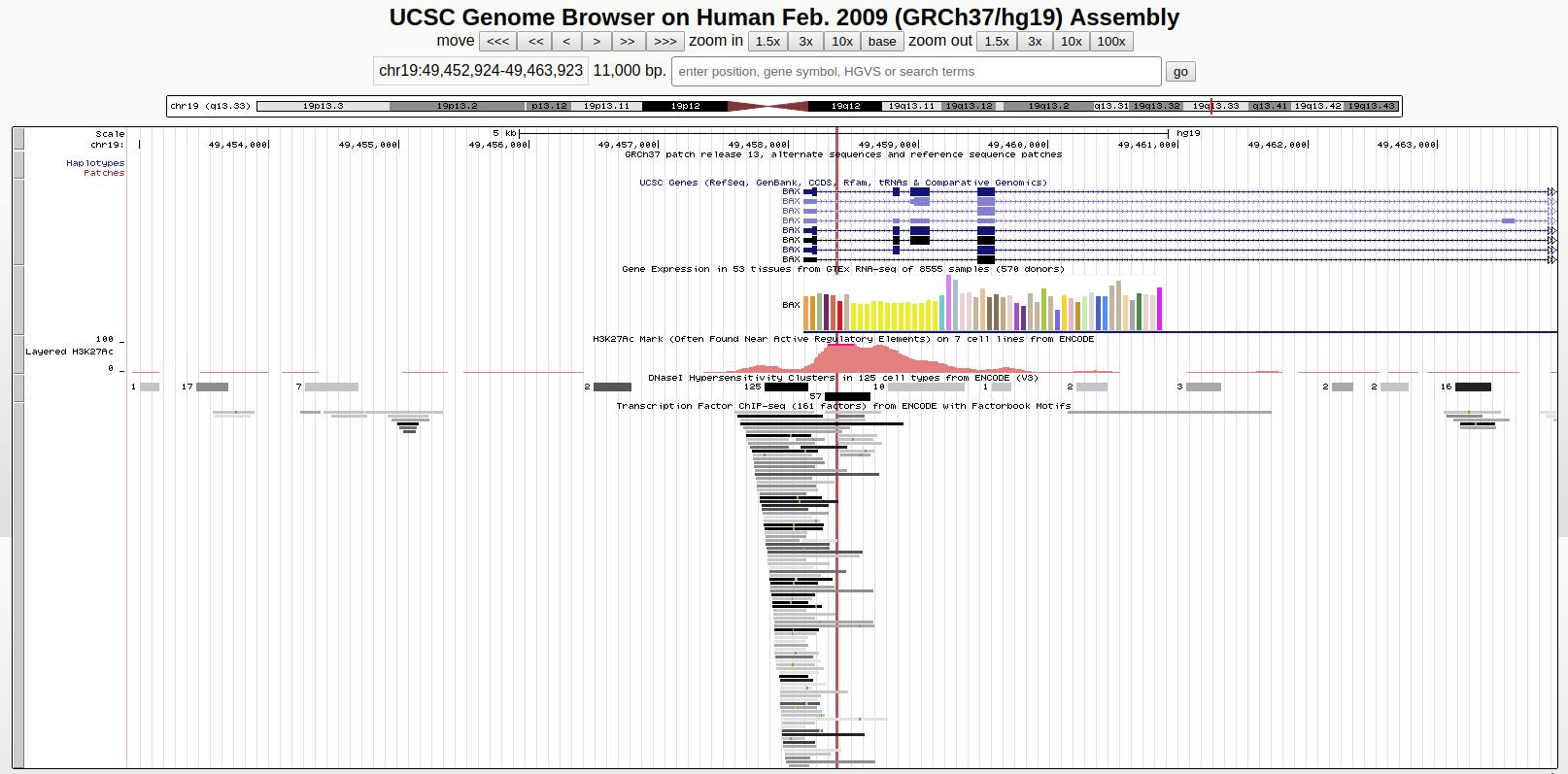


## NC_000010.10:g.91152143A>C in promoter of IFIT1


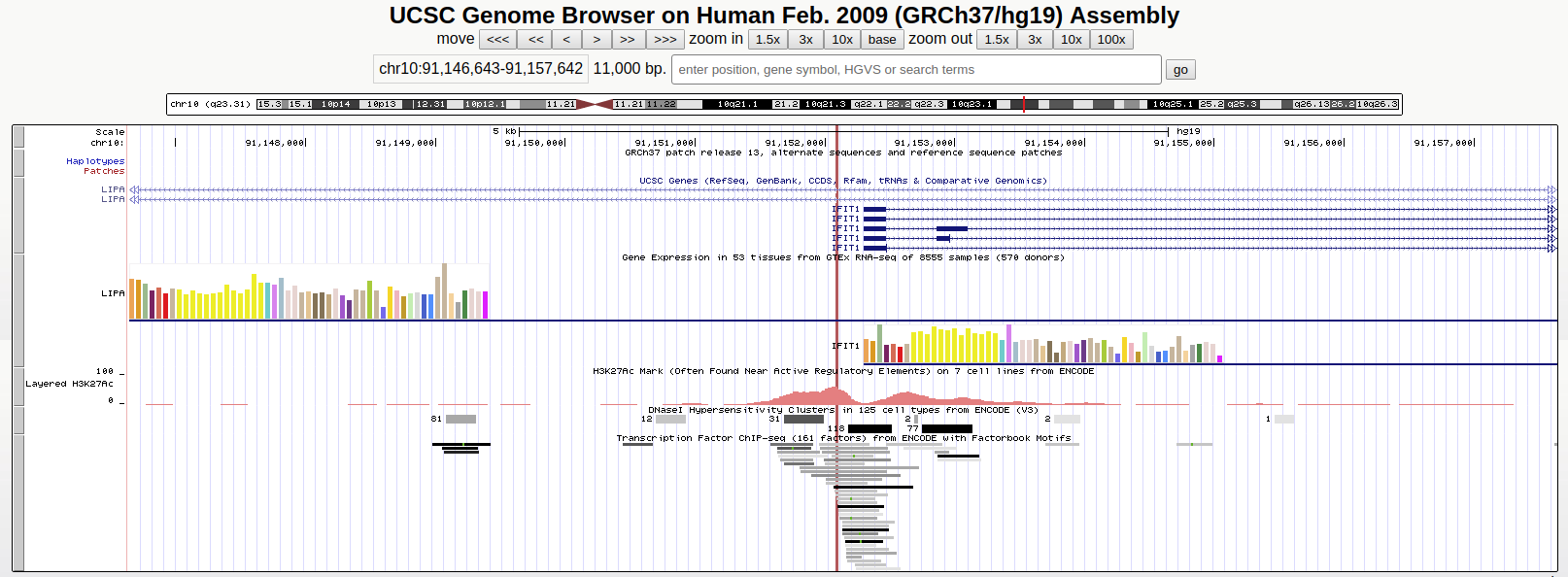


## NC_000014.8:g.95623820G>A in promoter of DICER


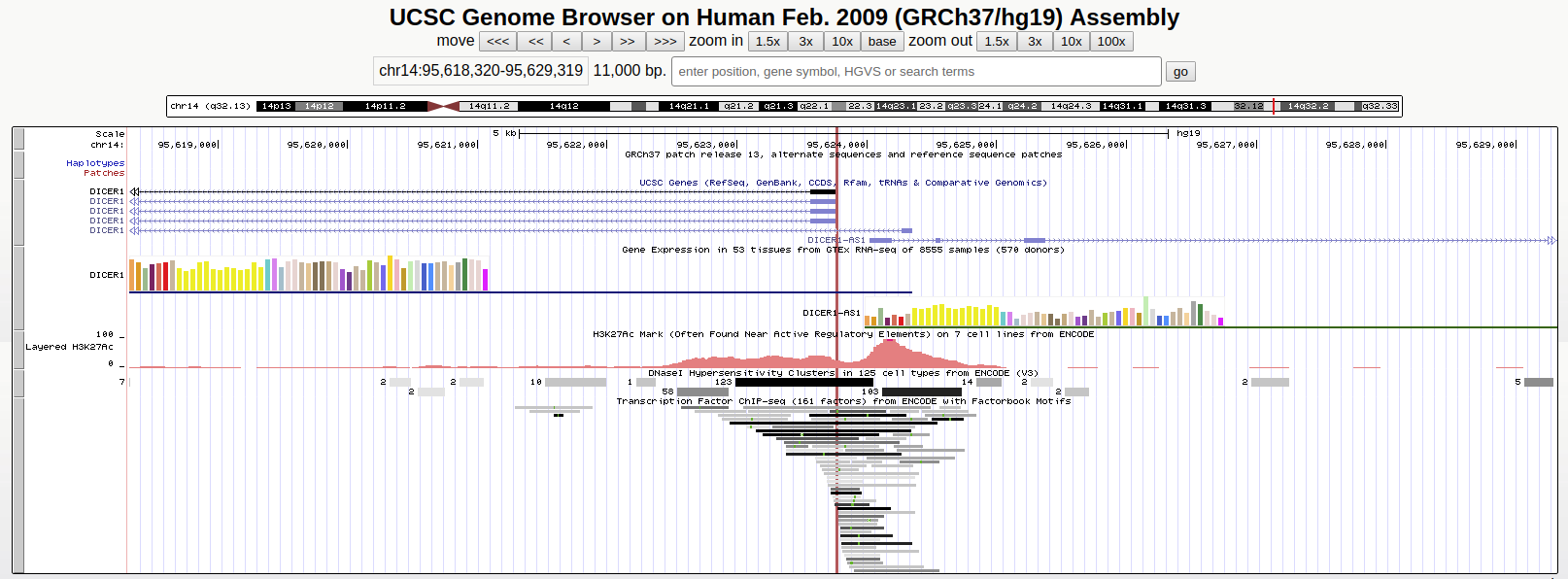


## NC_000005.9:g.179248888T>A in promoter of SQSTM1


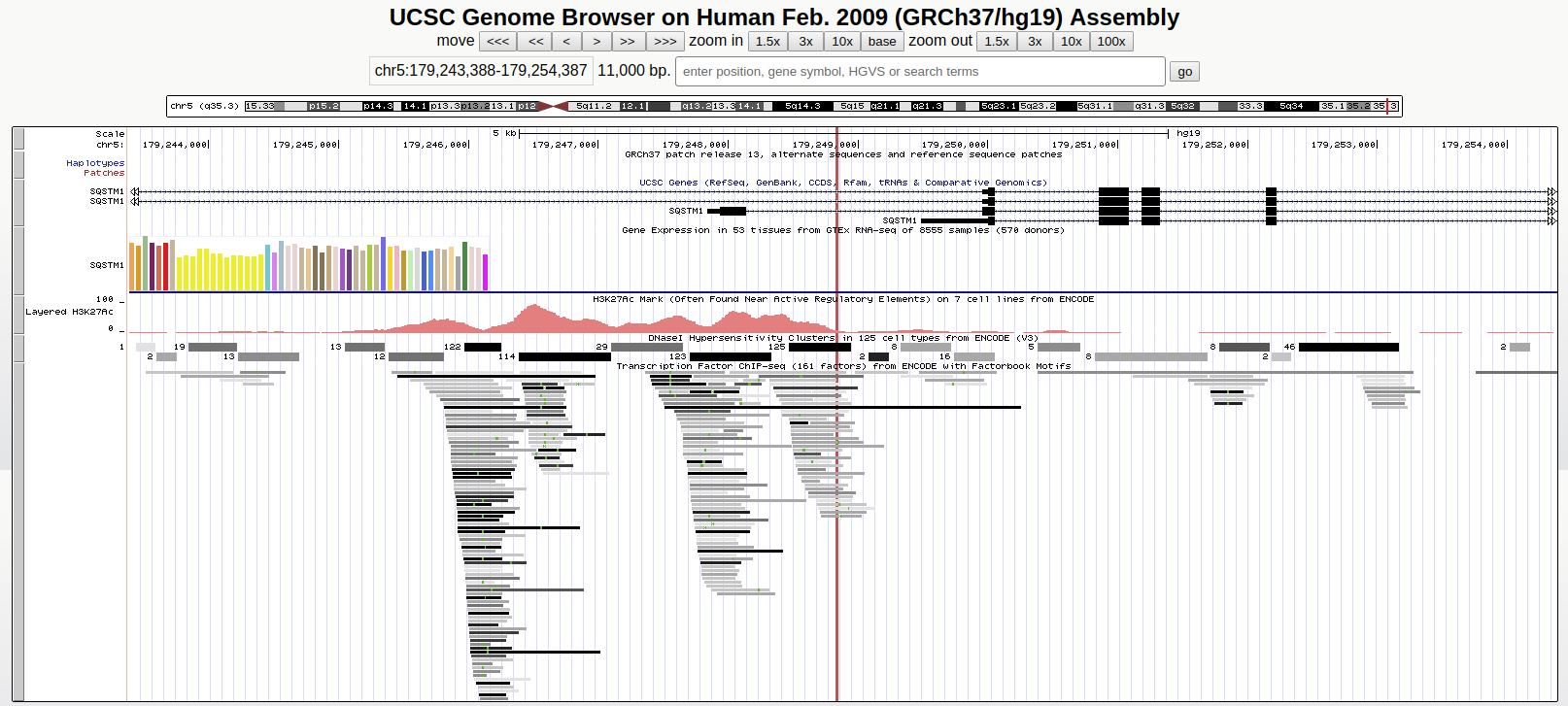


## NC_000004.11:g.56412867C>T in promoter of CLOCK


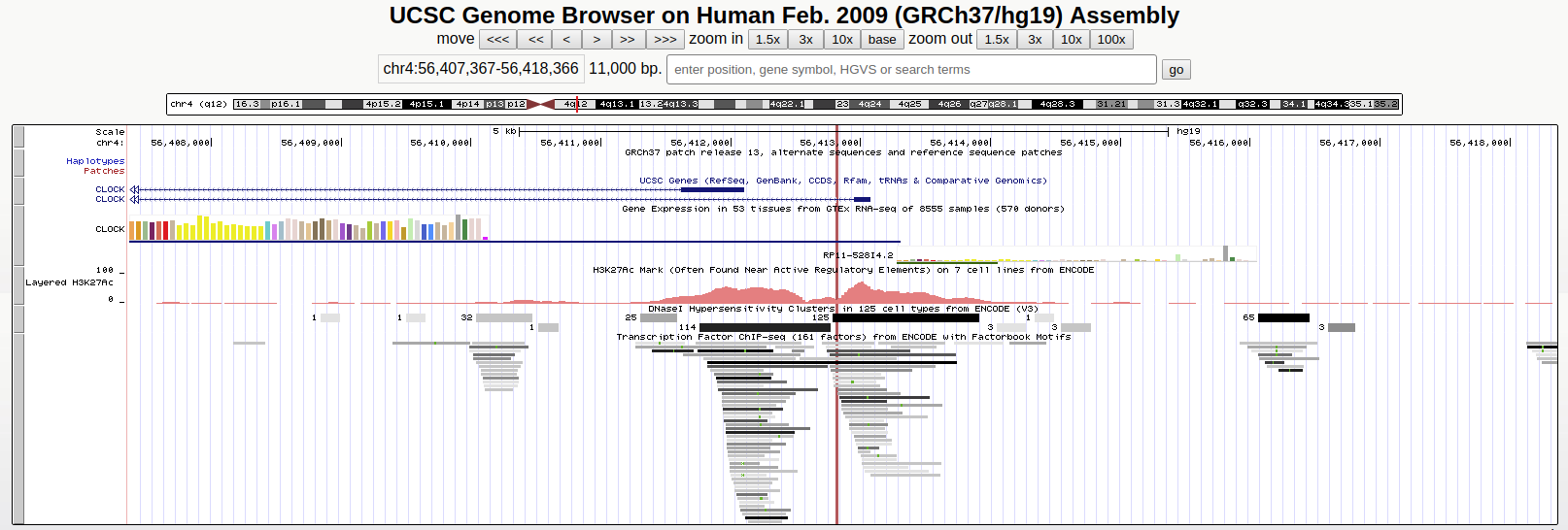


## NC_000019.9:g.661757C>T in promoter of RNF126


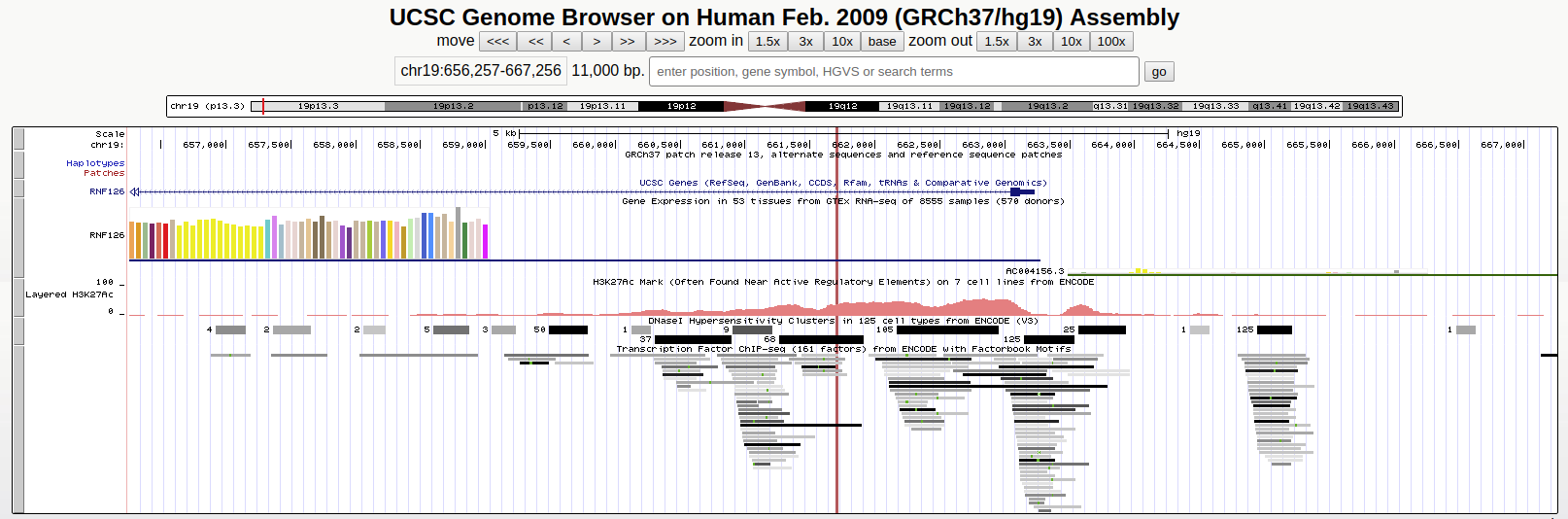

Supplement: Supplementary file 2 — Supplemental Figure S3 [file 41431_2020_698_MOESM2_ESM.docx]
