# Supplementary material for: Contributions of de novo variants to systemic lupus erythematosus
Source: Eur J Hum Genet. 2020 Jul 28;29(1):184–93. doi: 10.1038/s41431-020-0698-5 (PMC7852530; doi:10.1038/s41431-020-0698-5)

# Contributions of *de novo* variants to systemic lupus erythematosus

## Supplemental Figure S4

## European Journal of Human Genetics

Jonas Carlsson Almlöf^1*^, Sara Nystedt^1^, Aikaterini Mechtidou^1^, Dag Leonard^5^, Maija-Leena Eloranta^5^, Giorgia Grosso^4^, Christopher Sjöwall^2^, Anders A. Bengtsson^3^, Andreas Jönsen^3^, Iva Gunnarsson^4^, Elisabet Svenungsson^4^, Lars Rönnblom^5^, Johanna K. Sandling^5^, Ann-Christine Syvänen^1^

^1^Department of Medical Sciences, Molecular Medicine and Science for Life Laboratory, Uppsala University, 751 23 Uppsala, Sweden; ^2^Department of Clinical and Experimental Medicine, Rheumatology/Division of Neuro and Inflammation Sciences, Linköping University, 581 83 Linköping, Sweden; ^3^Department of Clinical Sciences, Rheumatology, Lund University, Skåne University Hospital, 222 42 Lund, Sweden; ^4^Department of Medicine, Karolinska Institutet, Rheumatology, Karolinska University Hospital, 171 77 Stockholm, Sweden; ^5^Department of Medical Sciences, Rheumatology and Science for Life Laboratory, Uppsala University, 751 85 Uppsala, Sweden;

## NC_000001.10:g.220445625C>T in promoter of RAB3GAP2


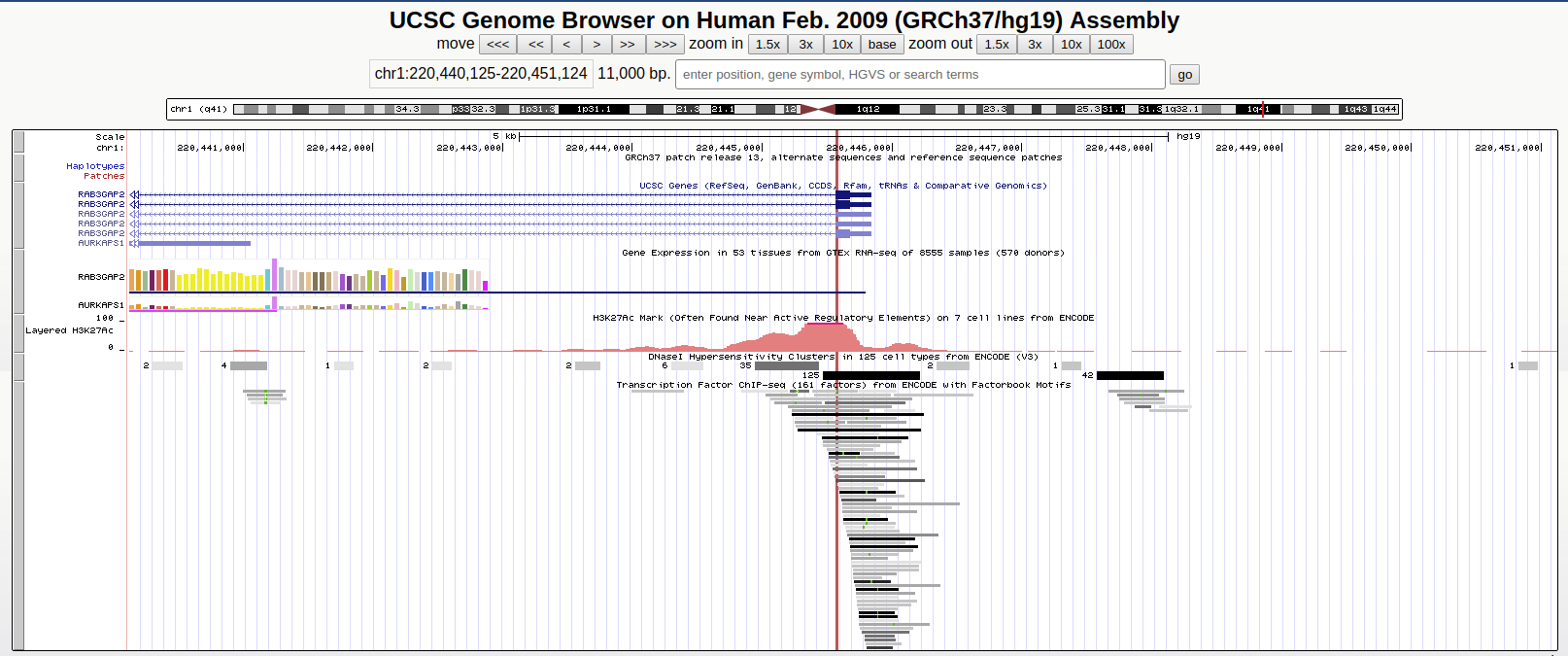


## NC_000006.11:g.5260643G>A in promoter of LYRM4


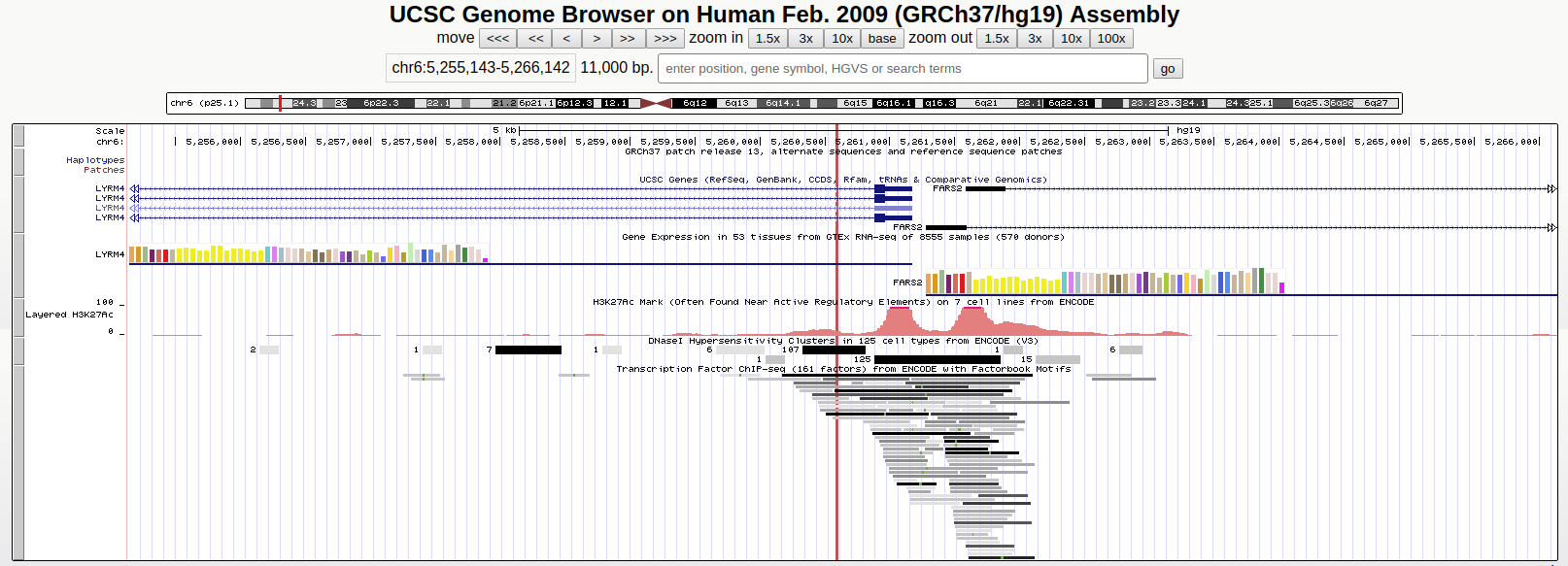


## NC_000006.11:g.87864999C>T in promoter of ZNF292


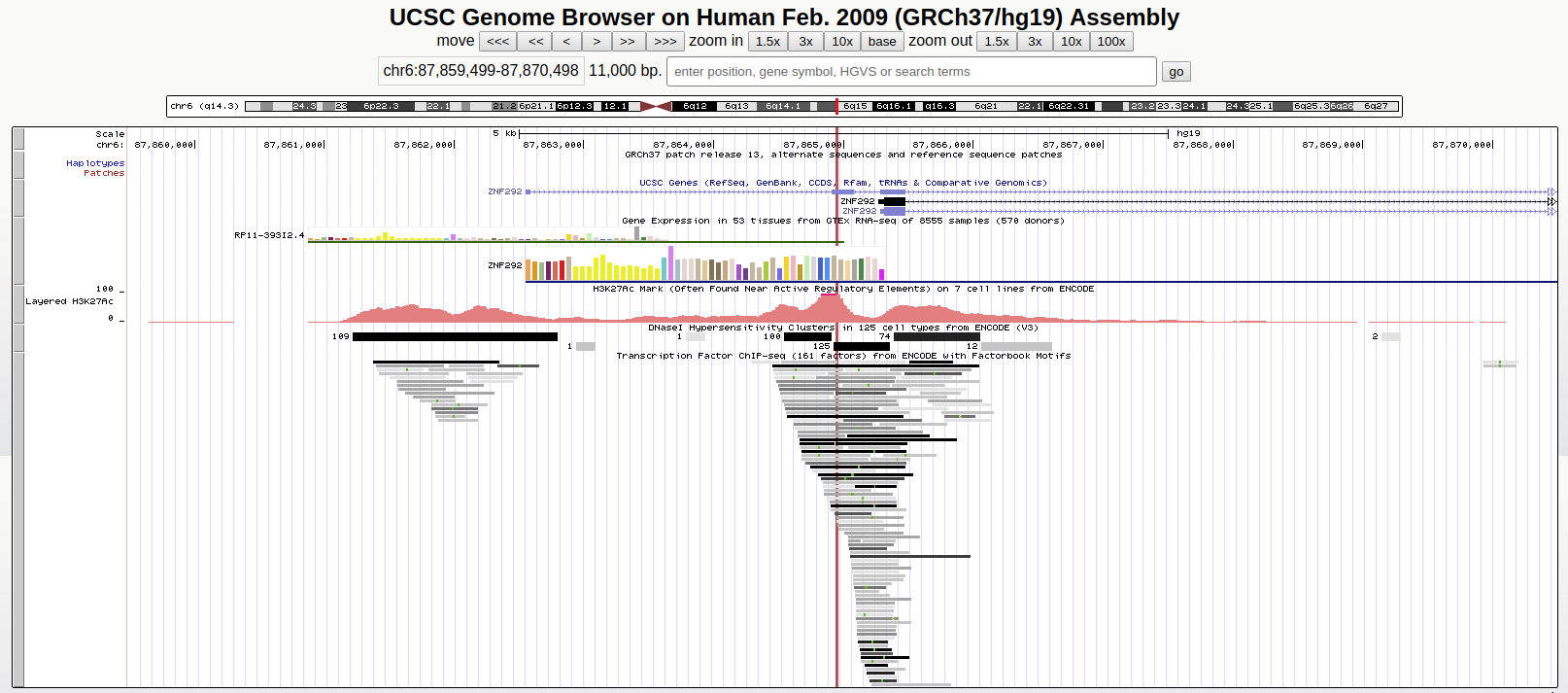


## NC_000007.13:g.106300401T>A in promoter of CCDC71L


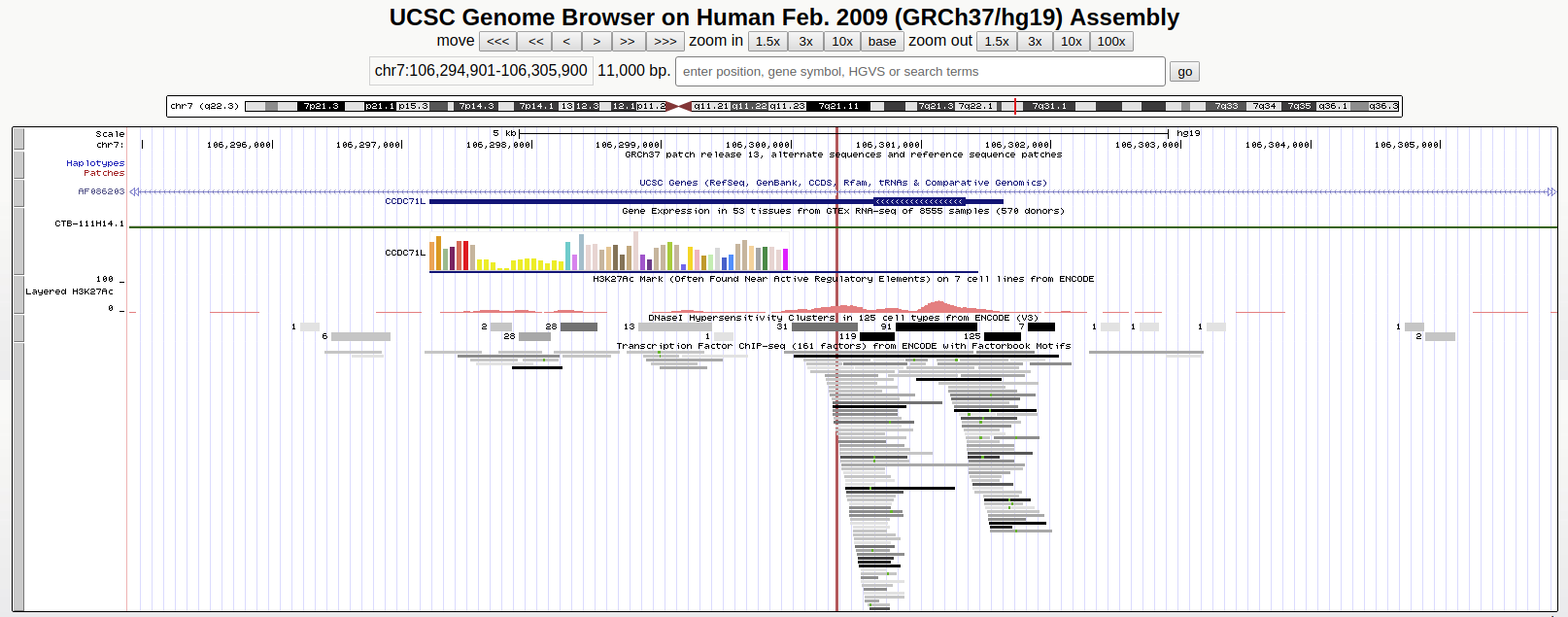


## NC_000013.10:g.77566039G>A in promoter of CLN5


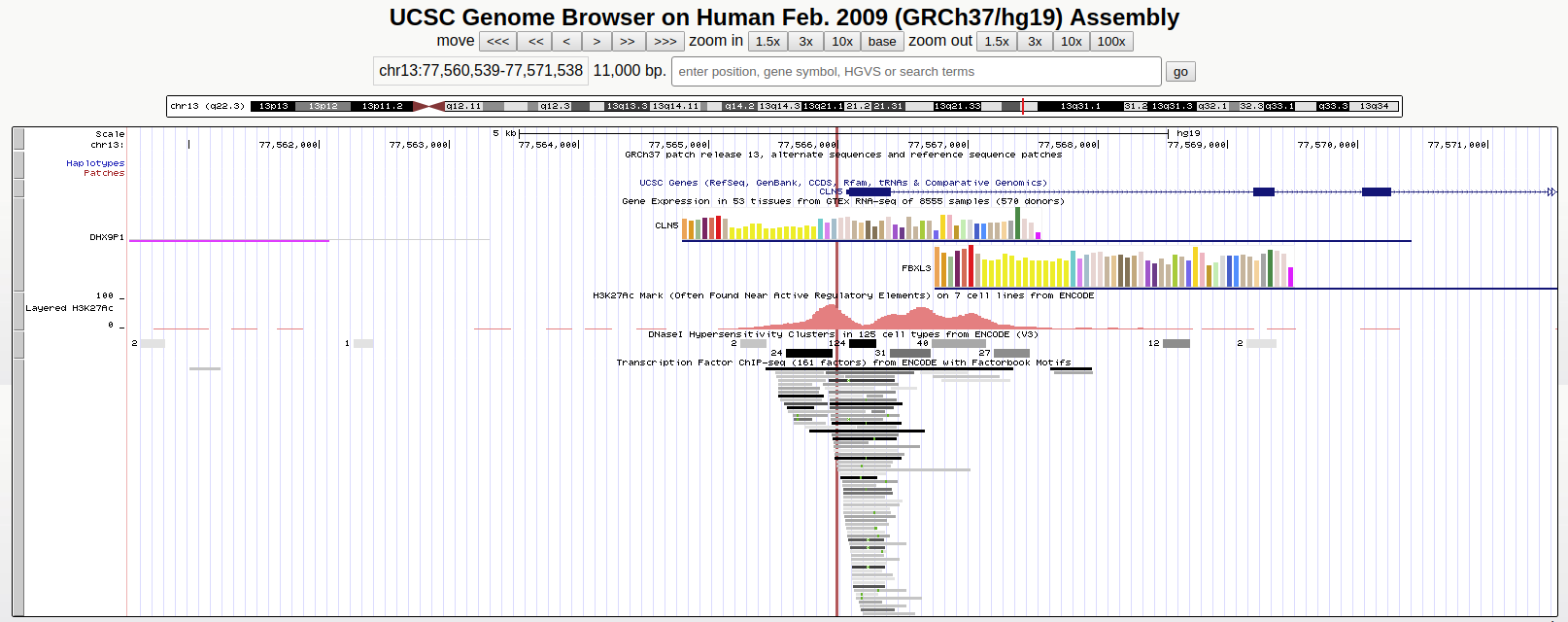


## NC_000016.9:g.56554091A>G in promoter of BBS2


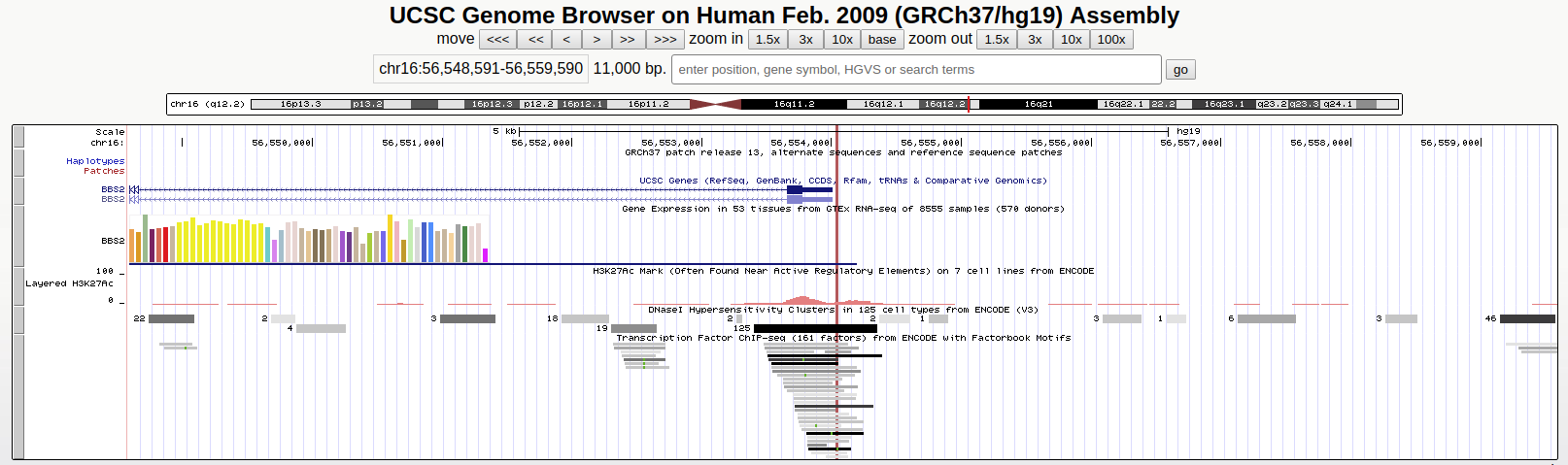


## NC_000017.10:g.60142354T>C in promoter of MED13


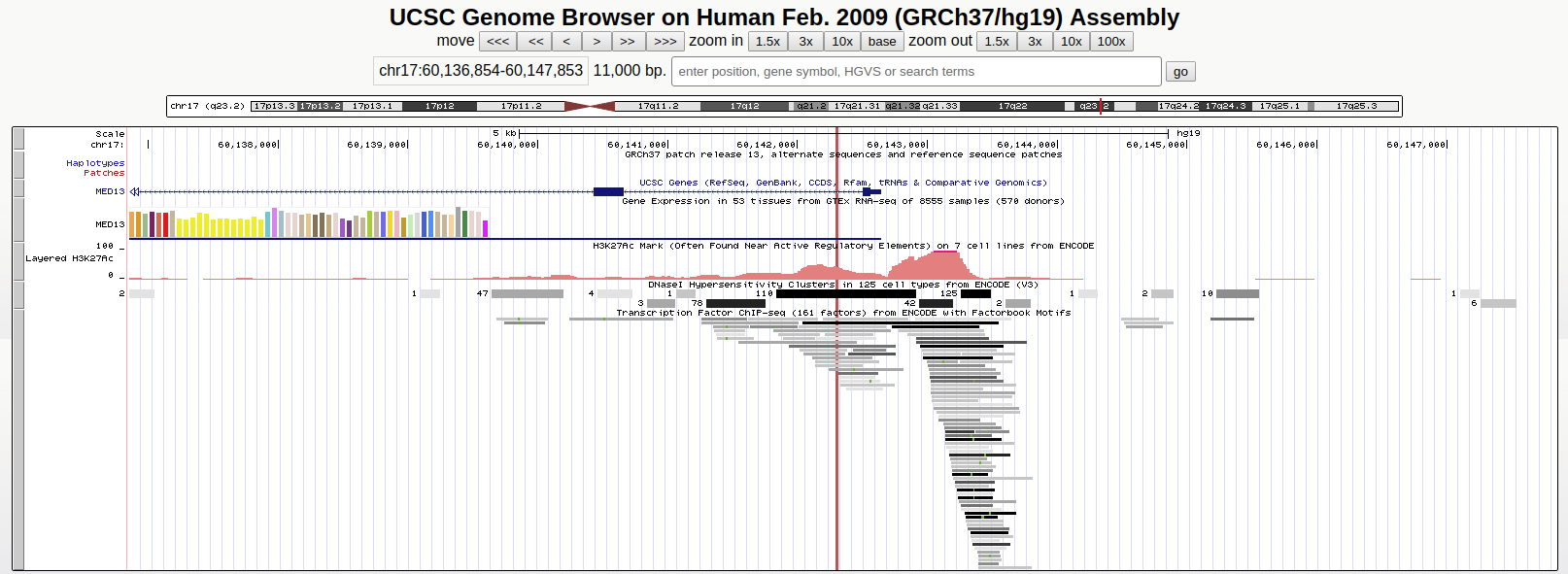


## NC_000018.9:g.12948527C>G in promoter of SEH1L


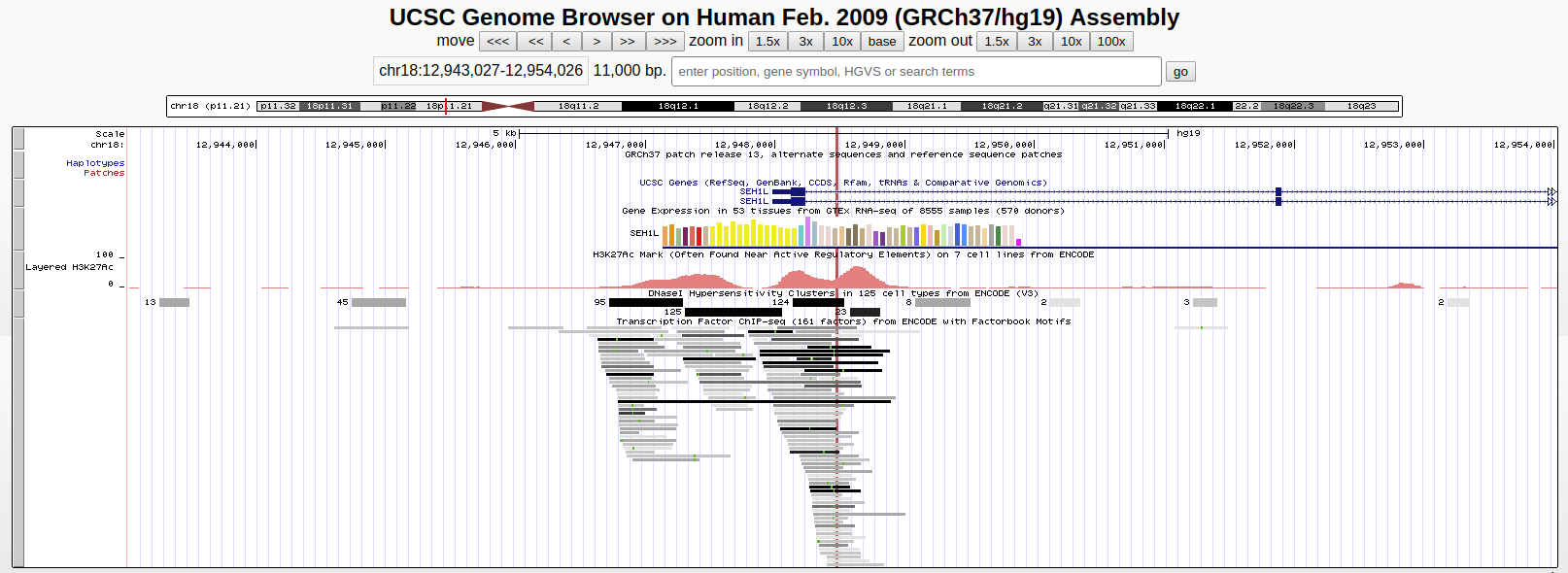


## NC_000003.11:g.44903329G>A in promoter of TMEM42


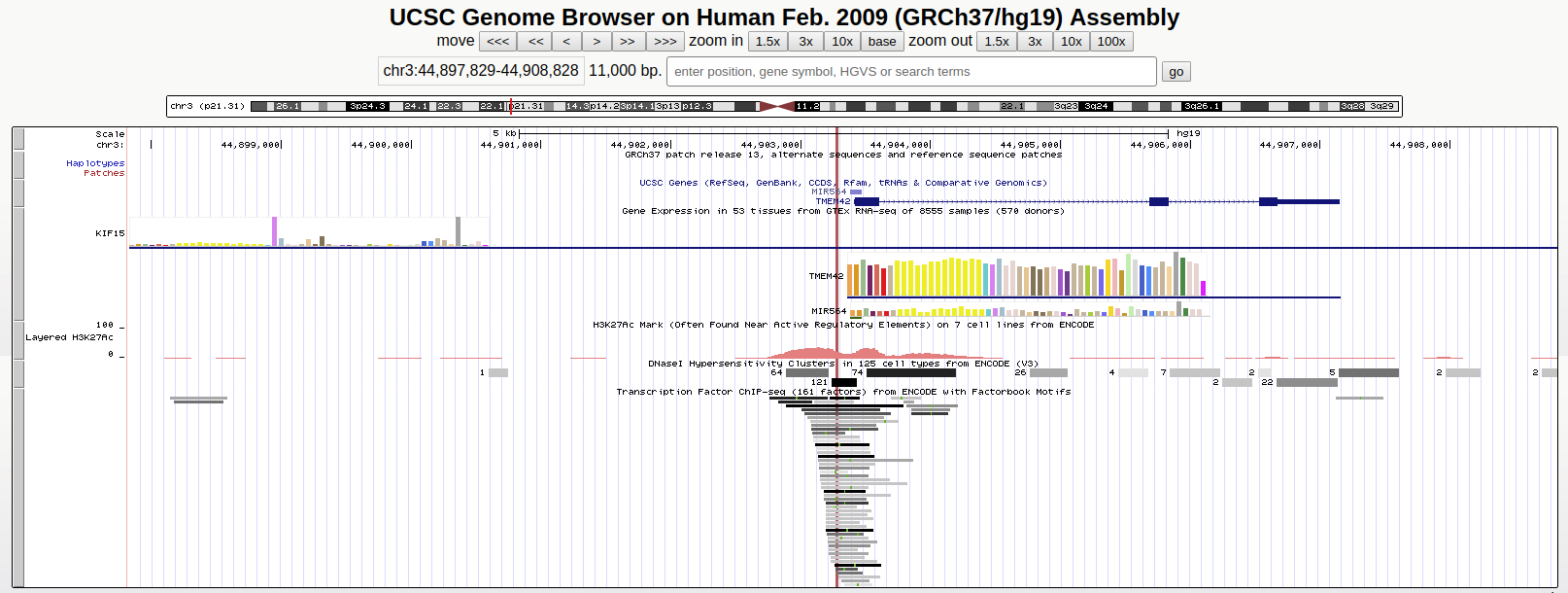

Supplement: Supplementary file 3 — Supplemental Figure S4 [file 41431_2020_698_MOESM3_ESM.docx]
