# Supplementary material for: Contributions of de novo variants to systemic lupus erythematosus
Source: Eur J Hum Genet. 2020 Jul 28;29(1):184–93. doi: 10.1038/s41431-020-0698-5 (PMC7852530; doi:10.1038/s41431-020-0698-5)
Supplement: Supplementary file 4 — Supplemental Table S2 [file 41431_2020_698_MOESM4_ESM.docx]

Supplementary Table S2 *De novo* SNVs with potential to increase the risk of SLE due to their location in promoters overlapping DNAse 1 hypersensitive sites

| **Gene** | **SNV position in genome^1^** | **No of TFBS in promotor, expression in blood cells^2,3,4^** | **FDR p-value for differential expression^5^** | **Genes with functional roles potentially relevant to SLE** |
| --- | --- | --- | --- | --- |
| *AIDA* | NC_000001.10: g.222886101A>G*^,m^ | 29 TFBS  High expression | 2.28E-12 | Inhibitor of JNK activation giving rise to organ damage in SLE. [1,2]. |
| *CLOCK* | NC_000004.11:  g.56412867C>T | 7 TFBS  High expression | 4.28E-13 | Daily variation of NF-κB immune response [3]. |
| *SQSTM1* | NC_000005.9:  g.179248888T>A*^,m^ | 13 TFBS  High expression | 6.96E-17 | Autophagy receptor involved in TLR7 and NOTCH1 signaling [4-6]. |
| *HIST1H1B* | NC_000006.11:  g.27835216G>A* | 19 TFBS  Over-expression | 0.000478 | Histone protein recognized by SLE autoantibodies [7]. |
| *DOCK8* | NC_000009.11: g.215166C>T^m^ | 29 TFBS  Over-expression | 1.56E-11 | Affects survival of B cells. Rare SLE-associated SNPs in gene. [8-10]. |
| *STOM* | NC_000009.11:  g.124132499C>A | 16 TFBS  Over-expression | 0.0000115 | Marker for distinguishing CD4+ Th1 from Th2 cells. Gene with Inflammatory role [11]. |
| *IFIT1* | NC_000010.10:  g.91152143A>C* | 5 TFBS  High expression | 4.41E-22 | Overexpressed in SLE patients [12]. |
| *CAND1* | NC_000012.11:  g.67663050G>A* | 25 TFBS  Expressed | 4.7E-09 | Reduces regulatory T cell functions [13,14]. |
| *SRP54* | NC_000014.8:  g.35452295G>A* | 20 TFBS  Over-expression | 8.35E-10 | Increased reactivity of SRP54 autoantigens against SLE. Associated with increased risk of stroke in SLE [15,16]. |
| *DICER* | NC_000014.8:  g.95623820G>A | 20 TFBS  Low expression (all tissues) | 0.00675 | Reduced in autoimmune MRL/lpr mouse model [17]. |
| *RNF126* | NC_000019.9:  g.661757C>T | 2 TFBS  Over-expression | 7.96E-08 | Class I MHC-mediated antigen presentation. |
| *BAX* | NC_000019.9:  49458424G>A* | 7 TFBS  Over-expression | 1.71E-12 | BAX is down-regulated in bone marrow cells from SLE patients and Bcl-2 to BAX ratio elevated in active SLE [18,19]. |

^1^Six of the positions are included in the dbSNP, but none has a minor allele frequency > 0.0004. ^2^All variants are annotated to promoters and DNase I hypersensitive sites. ^3^TFBS = Number of transcription factor binding sites in B cells. ^4^Expression levels refer to the highest expression in any blood cell type compared to other tissue types, where over-expression means blood tissue specific high expression and high expression means high expression in several tissues including blood tissues. ^5^Differential expression calculated using published gene expression omnibus (GEO) data from two studies on SLE patients and healthy controls [20,21]. *Validated by Sanger sequencing. ^m^Male patient. JNK = c-Jun N-terminal kinase

1. Rui Y, Xu Z, Xiong B, Cao Y, Lin S, Zhang M *et al*: A beta-catenin-independent dorsalization pathway activated by Axin/JNK signaling and antagonized by aida. Developmental cell. 2007; 13: 268-282.

2. Bloch O, Amit-Vazina M, Yona E, Molad Y, Rapoport MJ: Increased ERK and JNK activation and decreased ERK/JNK ratio are associated with long-term organ damage in patients with systemic lupus erythematosus. Rheumatology (Oxford). 2014; 53: 1034-1042.

3. Spengler ML, Kuropatwinski KK, Comas M, Gasparian AV, Fedtsova N, Gleiberman AS *et al*: Core circadian protein CLOCK is a positive regulator of NF-kappaB-mediated transcription. Proc Natl Acad Sci U S A. 2012; 109: E2457-2465.

4. Zhong Z, Umemura A, Sanchez-Lopez E, Liang S, Shalapour S, Wong J *et al*: NF-kappaB Restricts Inflammasome Activation via Elimination of Damaged Mitochondria. Cell. 2016; 164: 896-910.

5. Celhar T, Fairhurst AM: Toll-like receptors in systemic lupus erythematosus: potential for personalized treatment. Frontiers in pharmacology. 2014; 5: 265.

6. Li X, Liu F, Zhang X, Shi G, Ren J, Ji J *et al*: Notch-Hes-1 axis controls TLR7-mediated autophagic death of macrophage via induction of P62 in mice with lupus. Cell death & disease. 2016; 7: e2341.

7. Wesierska-Gadek J, Penner E, Lindner H, Hitchman E, Sauermann G: Autoantibodies against different histone H1 subtypes in systemic lupus erythematosus sera. Arthritis Rheum. 1990; 33: 1273-1278.

8. Randall KL, Lambe T, Johnson AL, Treanor B, Kucharska E, Domaschenz H *et al*: Dock8 mutations cripple B cell immunological synapses, germinal centers and long-lived antibody production. Nature immunology. 2009; 10: 1283-1291.

9. Wang M, Chen X, Zhang M, Zhu W, Cho K, Zhang H: Detecting significant single-nucleotide polymorphisms in a rheumatoid arthritis study using random forests. BMC proceedings. 2009; 3 Suppl 7: S69.

10. Martinez-Bueno M, Alarcon-Riquelme ME: Exploring Impact of Rare Variation in Systemic Lupus Erythematosus by a Genome Wide Imputation Approach. Frontiers in immunology. 2019; 10: 258.

11. Ono C, Yu Z, Kasahara Y, Kikuchi Y, Ishii N, Tomita H: Fluorescently activated cell sorting followed by microarray profiling of helper T cell subtypes from human peripheral blood. PLoS One. 2014; 9: e111405.

12. Ye S, Pang H, Gu YY, Hua J, Chen XG, Bao CD *et al*: Protein interaction for an interferon-inducible systemic lupus associated gene, IFIT1. Rheumatology (Oxford). 2003; 42: 1155-1163.

13. Pierce NW, Lee JE, Liu X, Sweredoski MJ, Graham RL, Larimore EA *et al*: Cand1 promotes assembly of new SCF complexes through dynamic exchange of F box proteins. Cell. 2013; 153: 206-215.

14. Ebner P, Versteeg GA, Ikeda F: Ubiquitin enzymes in the regulation of immune responses. Critical reviews in biochemistry and molecular biology. 2017; 52: 425-460.

15. Haddon DJ, Diep VK, Price JV, Limb C, Utz PJ, Balboni I: Autoantigen microarrays reveal autoantibodies associated with proliferative nephritis and active disease in pediatric systemic lupus erythematosus. Arthritis research & therapy. 2015; 17: 162.

16. Leonard D, Svenungsson E, Dahlqvist J, Alexsson A, Arlestig L, Taylor KE *et al*: Novel gene variants associated with cardiovascular disease in systemic lupus erythematosus and rheumatoid arthritis. Annals of the rheumatic diseases. 2018; 77: 1063-1069.

17. Divekar AA, Dubey S, Gangalum PR, Singh RR: Dicer insufficiency and microRNA-155 overexpression in lupus regulatory T cells: an apparent paradox in the setting of an inflammatory milieu. Journal of immunology. 2011; 186: 924-930.

18. Ohsako S, Hara M, Harigai M, Fukasawa C, S. K, Reed JC *et al*: The Bcl-2/Bax ratio of lymphocytes from human systemic lupus erythematosus patients. Japanese Journal of Rheumatology. 1997; 7: 305-313.

19. Alvarado-de la Barrera C, Alcocer-Varela J, Richaud-Patin Y, Alarcon-Segovia D, Llorente L: Differential oncogene and TNF-alpha mRNA expression in bone marrow cells from systemic lupus erythematosus patients. Scandinavian journal of immunology. 1998; 48: 551-556.

20. Petri M, Fu W, Ranger A, Allaire N, Cullen P, Magder LS *et al*: Association between changes in gene signatures expression and disease activity among patients with systemic lupus erythematosus. BMC medical genomics. 2019; 12: 4.

21. Banchereau R, Hong S, Cantarel B, Baldwin N, Baisch J, Edens M *et al*: Personalized Immunomonitoring Uncovers Molecular Networks that Stratify Lupus Patients. Cell. 2016; 165: 551-565.
