# Supplementary material for: Contributions of de novo variants to systemic lupus erythematosus
Source: Eur J Hum Genet. 2020 Jul 28;29(1):184–93. doi: 10.1038/s41431-020-0698-5 (PMC7852530; doi:10.1038/s41431-020-0698-5)
Supplement: Supplementary file 5 — Supplemental Table S4 [file 41431_2020_698_MOESM5_ESM.docx]

Supplemental Table S4 *De novo* non-synonymous SNVs with potential to affect SLE risk.

| **Gene** | **Position (amino acid change, transcript annotation, and genomic coordinate)** | **Potential effect on protein** | **Blood cell type with reported highest expression** | **FDR p-value for differential expression^1^** | **Function potentially relevant to SLE** |
| --- | --- | --- | --- | --- | --- |
| *MAZ* | P56270-1:  p.(Cys368Gly) ENST00000322945.11: c.1275T>G  NC_000016.9:  g.29819609T>G* | Disrupts zinc binding in C2H2-type zinc finger. | PBMC, NK | 9.40E-07 | Regulates inflammation-responsive genes [1]. |
| *LTB4R2* | Q9NPC1-1:  p.(Gln169*) ENST00000533293.2: c.631C>T  NC_000014.8:  g.24780375C>T | Truncated protein (4 transmembrane helices missing). | PBMC | 0.301 | Reduced mRNA expression in CD4(+) T cells in asthma patients [2]. |
| *ISX* | Q2M1V0-1:  p.(Arg138Gln) ENST00000308700.6: c.1365G>A  NC_000022.10:  g.35480407G>A* | Disrupts DNA binding in the major DNA groove. | - | 0.929 | Regulates Vitamin A which is reduced in SLE patients [3,4]. |

^1^Differential expression calculated using published gene expression omnibus (GEO) data from two studies on SLE patients and healthy controls [5,6]. *Validated by Sanger sequencing.

1. Ray A, Dhar S, Shakya A, Ray P, Okada Y, Ray BK: SAF-3, a novel splice variant of the SAF-1/MAZ/Pur-1 family, is expressed during inflammation. The FEBS journal. 2009; 276: 4276-4286.

2. Matsunaga Y, Fukuyama S, Okuno T, Sasaki F, Matsunobu T, Asai Y *et al*: Leukotriene B4 receptor BLT2 negatively regulates allergic airway eosinophilia. FASEB journal : official publication of the Federation of American Societies for Experimental Biology. 2013; 27: 3306-3314.

3. Lobo GP, Amengual J, Baus D, Shivdasani RA, Taylor D, von Lintig J: Genetics and diet regulate vitamin A production via the homeobox transcription factor ISX. The Journal of biological chemistry. 2013; 288: 9017-9027.

4. Handono K, Firdausi SN, Pratama MZ, Endharti AT, Kalim H: Vitamin A improve Th17 and Treg regulation in systemic lupus erythematosus. Clinical rheumatology. 2016; 35: 631-638.

5. Banchereau R, Hong S, Cantarel B, Baldwin N, Baisch J, Edens M *et al*: Personalized Immunomonitoring Uncovers Molecular Networks that Stratify Lupus Patients. Cell. 2016; 165: 551-565.

6. Petri M, Fu W, Ranger A, Allaire N, Cullen P, Magder LS *et al*: Association between changes in gene signatures expression and disease activity among patients with systemic lupus erythematosus. BMC medical genomics. 2019; 12: 4.
