# Supplementary material for: Contributions of de novo variants to systemic lupus erythematosus
Source: Eur J Hum Genet. 2020 Jul 28;29(1):184–93. doi: 10.1038/s41431-020-0698-5 (PMC7852530; doi:10.1038/s41431-020-0698-5)
Supplement: Supplementary file 6 — Supplemental Table S6 [file 41431_2020_698_MOESM6_ESM.docx]

Supplemental Table S6 *De novo* structural variants with potential to affect SLE

| **Gene** | **Structural variant position** | **Length (bp)** | **Genomic effect** | **Gene function potentially relevant to SLE** |
| --- | --- | --- | --- | --- |
| *RBM10* | NC_000023.10:g.47017419_47037305del | 19,887 | Deletion of exons 3-7 | Regulates the activity of NF-κB-responsive promoters and consequently inflammation development [1]. |
| *SMARCA2* | NC_000009.11:g.2194227_2197614del | 3,388 | Deletion 603 bases downstream | Downregulated in SLE patients with the MECP2 risk haplotype. The patient has the protective haplotype and high expression of SMARCA2 [2]. |
| *PPARA* | NC_000022.10:g.46625870_46626103del | 234 | Deletion 80kb from TSS in intron 6 | Regulates differentiation of T cells and contributes to the regulating of the activity of NF-KappaB [3,4]. |

TSS = transcription start site

1. Atsumi T, Suzuki H, Jiang JJ, Okuyama Y, Nakagawa I, Ota M *et al*: Rbm10 regulates inflammation development via alternative splicing of Dnmt3b. International immunology. 2017; 29: 581-591.

2. Webb R, Wren JD, Jeffries M, Kelly JA, Kaufman KM, Tang Y *et al*: Variants within MECP2, a key transcription regulator, are associated with increased susceptibility to lupus and differential gene expression in patients with systemic lupus erythematosus. Arthritis Rheum. 2009; 60: 1076-1084.

3. Yang Y, Gocke AR, Lovett-Racke A, Drew PD, Racke MK: PPAR Alpha Regulation of the Immune Response and Autoimmune Encephalomyelitis. PPAR research. 2008; 2008: 546753.

4. Vanden Berghe W, Vermeulen L, Delerive P, De Bosscher K, Staels B, Haegeman G: A paradigm for gene regulation: inflammation, NF-kappaB and PPAR. Advances in experimental medicine and biology. 2003; 544: 181-196.
