# Supplementary material for: Contributions of de novo variants to systemic lupus erythematosus
Source: Eur J Hum Genet. 2020 Jul 28;29(1):184–93. doi: 10.1038/s41431-020-0698-5 (PMC7852530; doi:10.1038/s41431-020-0698-5)
Supplement: Supplementary file 8 — Supplemental Material and Results [file 41431_2020_698_MOESM8_ESM.docx]

# Contributions of *de novo* variants to systemic lupus erythematosus

## European Journal of Human Genetics

Jonas Carlsson Almlöf^1*^, Sara Nystedt^1^, Aikaterini Mechtidou^1^, Dag Leonard^5^, Maija-Leena Eloranta^5^, Giorgia Grosso^4^, Christopher Sjöwall^2^, Anders A. Bengtsson^3^, Andreas Jönsen^3^, Iva Gunnarsson^4^, Elisabet Svenungsson^4^, Lars Rönnblom^5^, Johanna K. Sandling^5^, Ann-Christine Syvänen^1^

^1^Department of Medical Sciences, Molecular Medicine and Science for Life Laboratory, Uppsala University, 751 23 Uppsala, Sweden; ^2^Department of Clinical and Experimental Medicine, Rheumatology/Division of Neuro and Inflammation Sciences, Linköping University, 581 83 Linköping, Sweden; ^3^Department of Clinical Sciences, Rheumatology, Lund University, Skåne University Hospital, 222 42 Lund, Sweden; ^4^Department of Medicine, Karolinska Institutet, Rheumatology, Karolinska University Hospital, 171 77 Stockholm, Sweden; ^5^Department of Medical Sciences, Rheumatology and Science for Life Laboratory, Uppsala University, 751 85 Uppsala, Sweden;

# Supplemental Methods and Results

## Calling and filtering of *de novo* INDELs

*De novo* INDEL candidates were called using two programs: GATK version 3.5.0 and Triodenovo ([Wei et al., 2015](#_ENREF_5)) version 0.04. By GATK *de novo* INDELs were called with the VariantAnnotator using --annotation PossibleDeNovo and Triodenovo was run with default parameters.

The raw *de novo* INDEL calls were discarded if they fulfilled any of the following ten criteria:
- INDELs located on the Y chromosome, in un-localized contigs or alternative haplotypes.
- INDELs that over-lapped with *de novo* INDELs in any other sample.
- INDELs with supporting reads in any of the parents.
- INDELS with allelic imbalance greater than 0.7.
- INDELs with average read depth below 15.
- INDELs not annotated with PASS by the GATK calling quality filtering.
- INDELs marked as a low confidence *de novo* INDEL by GATK.
- INDELs flanked by at least one additional *de novo* INDEL or at least three additional *de novo* SNVs within 100bp.
- INDELs that overlapped with the ENCODE blacklisted regions ([Dunham et al., 2012](#_ENREF_4)), representing regions with frequent artifacts in next generation sequencing.
- INDELs that overlapped a segmental duplication ([Bailey et al., 2002](#_ENREF_1); [Bailey, Yavor, Massa, Trask, & Eichler, 2001](#_ENREF_2)), including regions of at least 1kb that were more than 90% similar to another region in the genome.

Additionally, the INDELs were annotated with overlapping repeats found in the RepeatMasker track in the USCS browser [developed by A.F.A. Smit, R. Hubley, and P. Green, http://www.repeatmasker.org/] and CRG mappability scores generated using 100-mers with no more than two mismatches ([Derrien et al., 2012](#_ENREF_3)). Based on the additional annotations the INDELs were further manually filtered.

Calling and stringent filtering of *de novo* INDELs resulted in 309 candidate *de novo* INDELs. Per individual this equal 4.3 INDELs which is similar to the number of *de novo* INDELs found in deCODE per individual. The candidates were further investigated based on annotations and literature search of the nearby genes followed by manual inspection in IGV. The vast majority of the candidate *de novo* INDELs where deemed to be non-functional because they were situated far away from any genes in a genomic region without any functional elements. In the end, 5 *de novo* INDELs where found possibly affecting nearby genes potentially relevant to SLE, see Supplemental Table S3.

## References

Bailey, J. A., Gu, Z., Clark, R. A., Reinert, K., Samonte, R. V., Schwartz, S., . . . Eichler, E. E. (2002). Recent segmental duplications in the human genome. *Science, 297*(5583), 1003-1007. doi: 10.1126/science.1072047

Bailey, J. A., Yavor, A. M., Massa, H. F., Trask, B. J., & Eichler, E. E. (2001). Segmental duplications: organization and impact within the current human genome project assembly. *Genome Res, 11*(6), 1005-1017. doi: 10.1101/gr.187101

Derrien, T., Estelle, J., Marco Sola, S., Knowles, D. G., Raineri, E., Guigo, R., & Ribeca, P. (2012). Fast computation and applications of genome mappability. *PLoS One, 7*(1), e30377. doi: 10.1371/journal.pone.0030377

Dunham, I., Kundaje, A., Aldred, S. F., Collins, P. J., Davis, C. A., Doyle, F., . . . Lochovsky, L. (2012). An integrated encyclopedia of DNA elements in the human genome. *Nature, 489*(7414), 57-74. doi: nature11247

Wei, Q., Zhan, X., Zhong, X., Liu, Y., Han, Y., Chen, W., & Li, B. (2015). A Bayesian framework for de novo mutation calling in parents-offspring trios. *Bioinformatics, 31*(9), 1375-1381. doi: 10.1093/bioinformatics/btu839
